# Prognostic Significance and Immune Correlation of CCL3 Expression in Colon Adenocarcinoma: Insights From Multidatabase Analysis

**DOI:** 10.1155/bri/6647501

**Published:** 2026-04-17

**Authors:** Vikrant Kumar, Nawaid Hussain Khan, Shashi Nandar Kumar, Neeraj Mahajan, Wafa Aziz, Vinay Kumar, Chaitenya Verma

**Affiliations:** ^1^ Department of Biophysics, All India Institute of Medical Sciences (AIIMS), New Delhi, 29, India, aiims.edu; ^2^ Faculty of Medicine, Ala-Too International University, Bishkek, Kyrgyzstan; ^3^ Department of Occupational and Environmental Health, Faculty of Pharmaceutical Sciences, Tokyo University of Science, Noda, 278-8510, Japan, tus.ac.jp; ^4^ Department of Microbiology, All India Institute of Medical Sciences (AIIMS), New Delhi, 29, India, aiims.edu; ^5^ Pennsylvania State University Hershey Medical Center, 500 University Dr, Hershey, Pennsylvania, USA; ^6^ Department of Biotechnology, School of Engineering and Technology, Sharda University, Grater Noida, India, sharda.ac.in

**Keywords:** CCL3, colon adenocarcinoma, immune infiltration, miRNA, prognostic marker

## Abstract

**Objective:**

Colon adenocarcinoma (COAD) remains a leading cause of cancer‐related mortality, necessitating reliable prognostic biomarkers. This study explored the potential prognostic role of C‐C motif chemokine ligand 3 (CCL3) and its association with immune‐related signatures in COAD.

**Methods:**

Multiple publicly accessible databases, including TCGA, GEPIA2, UALCAN, TIMER, and Kaplan–Meier (KM) plotter, were used to examine the expression and prognostic profiles of CCL3 in various cancers. The association between CCL3 expression and clinical stage, immune infiltration, and immune gene markers was investigated using TIMER, GEPIA2, and TISIDB databases. Furthermore, we conducted functional enrichment analyses (GO and KEGG) of genes coexpressed with CCL3, assessed methylation status using OncoBD and MEXPRESS, and developed a gene–miRNA interaction network using miRWalk to elucidate their potential roles in COAD.

**Results:**

The expression of CCL3 was significantly upregulated in COAD and most other malignancies. Furthermore, high CCL3 expression was significantly associated with poor overall survival (OS) and relapse‐free survival (RFS), with a univariate HR = 1.52 (95% CI: 1.12–2.06, *p* < 0.01). Multivariate Cox regression confirmed CCL3 as an independent prognostic factor after adjusting for the TNM stage, age, gender, and grade (adjusted HR = 1.42, 95% CI: 1.05–1.92, *p* = 0.023). CCL3 showed strong positive correlations with immune cell infiltration, particularly macrophages (Cor = 0.72, *p* < 0.001) and neutrophils (Cor = 0.74, *p* < 0.001), suggesting its role in shaping an immunosuppressive tumor immune microenvironment (TIME). Additionally, preliminary correlations indicate that high CCL3 may be associated with chemotherapy resistance.

**Conclusion:**

This study suggests that CCL3 could serve as a potential prognostic marker for COAD and other cancers. Because CCL3 expression was strongly associated with immune modulation within the tumor microenvironment, this pointed out that CCL3 could serve as a promising therapeutic target with significant implications for immunotherapy and chemotherapy response.

## 1. Introduction

Colorectal cancer ranks as the third most frequent diagnosis globally, with a fatality rate second only to lung cancer [1]. According to estimates from the Surveillance, Epidemiology, and End Results (SEER) database, more than 153,000 new cases of colorectal cancer were detected in 2023, accounting for 7.8% of all new cancer cases [2]. The majority of colon cancers are now diagnosed asymptomatically because of the development of screening colonoscopies. The location and size of the tumor determine the symptoms and their intensity. Rectal bleeding (37%), stomach pain (34%), and anemia (23%) are common symptoms [3]. The standard therapy for locally resectable colon cancer is surgical resection. Tumor location and stage determine the type of resection, extent of lymphadenectomy, and specific procedures [4]. In certain cases of advanced colon cancer, adjuvant chemotherapy is recommended [5]. Systemic therapy is used to treat patients with metastatic disease. Radiation is rarely employed in colon cancer cases [6]. The survival rate for patients with COAD is poor despite advancements in early detection and therapy, as many patients are diagnosed at an advanced stage. In addition, the development of drug resistance is a challenge for current treatment options, such as chemotherapy, radiation, and targeted therapy. Hence, identifying new molecular targets can provide alternative or complementary treatment strategies, especially for patients who do not respond to existing therapy. However, approximately 70% of these cancers are colonic, while rectal tumors comprise the remaining 30% [2]. More than 90% of colon cancer cases are adenocarcinomas, with the remainder being lymphomas, neuroendocrine tumors, and gastrointestinal stromal tumors [7]. The behavior of tumors is influenced by abilities of cancer cells and the surrounding tumor microenvironment (TME), both of which play direct roles in tumor growth, invasion, and metastasis [8]. Tumor cells interact with their supportive stroma by releasing angiogenic factors, integrins, proteases, cytokines, and chemokines [8, 9]. Chemokines can exert antitumor effects by attracting immune effector cells to the TME [10]. However, growing evidence indicates that chemokines may also promote tumorigenesis in various cancers [9, 10], including oral squamous cell carcinoma (OSCC) [11]. The tumor‐promoting roles of chemokines involve inducing the migration of inflammatory cells, enhancing the motility of cancerous cells, promoting new blood vessel formation, and remodeling the extracellular matrix [8–13].

CCL3 belongs to the C‐C motif family of chemokines. It is also known as macrophage inflammatory protein‐1*α* (MIP‐1*α*) and plays a role in inflammation [14]. A key modulator of the immunological milieu, CCL3 plays a key role in immune cell trafficking in cancer and inflammation. It modulates several immune cell subtypes by binding to three different receptors: CCR1 on monocytes and myeloid cells, CCR4 on Th2 T cells and Tregs, and CCR5 on Th1 T cells, NK cells, and plasmacytoid dendritic cells (DCs). [15, 16]. CCL3 plays a multifaceted role in tumor cell chemoattractiveness by regulating T‐cell trafficking and attracting immunosuppressive cells to the tumor beds. Increased infiltration of tumor‐related macrophages, regulatory T cells, and myeloid‐derived suppressor cells (MDSCs) is associated with elevated CCL3 levels, which enable tumor cells to evade immune surveillance. [17–19]. CCL3 has become a crucial biomarker for immune activation and response, with high serum levels associated with poor prognosis in patients with colorectal cancer [20]. High CCL3 plasma levels are linked to an increased risk of progression in chronic lymphocytic leukemia (CLL) [21], decreased progression‐free survival in diffuse large B‐cell lymphoma (DLBCL) [22], shorter progression‐free survival and aberrant bone remodeling in Waldenström’s macroglobulinemia [23], and decreased survival in multiple myeloma [24]. As previously shown, CCL3 is highly expressed in tumor‐associated macrophages (TAMs) and cancer cells in colorectal adenocarcinoma (COAD). This triggers the Akt signaling pathway by interacting with CCR5. The CCL3–CCR5 axis enhances the migration and invasion abilities of COAD cells by activating AKT signaling. CCL3, derived from both TAMs and cancer cells, plays a role in the malignant traits of colorectal cancer (COAD). High levels of CCL3/CCR5 expression are strongly associated with poor prognosis in patients with COAD [25]. Although CCL3 is known to play a role in inflammatory processes, its impact on the progression of colorectal cancer is not well understood. To address this gap, the present study utilized an integrated bioinformatic analysis of multiple databases to investigate the differential expression, prognostic signature, and potential biological roles of CCL3 in various cancer types, with a specific emphasis on COAD. Additionally, we investigated the association between CCL3 expression and tumor immune infiltration (TII), methylation status, and miRNA regulatory networks. Therefore, this study may provide new evidence for understanding the role of CCL3 in COAD in the context of prognosis and as a possible therapeutic target.

## 2. Materials and Methods

### 2.1. Analysis Utilizing GEPIA2, UALCAN, and TIMER Databases

The relative expression of CCL3 was analyzed using three distinct datasets. Data were retrieved from TCGA‐COAD (*n* = 459 tumors, *n* = 41 normal samples) and analyzed using GEPIA2 for expression and correlations. The GEPIA2 database contains mRNA expression data from 9736 tumors and 8587 normal tissue samples [26]. The GEPIA2 database was used to explore the expression of CCL3 in the TCGA pan‐cancer cohort in tumors and TCGA combined with GTEx samples, with a log_2_FC cutoff of 1 and a *p* value cutoff of 0.01. The UALCAN database is an interactive online resource that offers an analysis of cancer omics data for 14 different cancer types [27]. The UALCAN database used data from the TCGA cohort to analyze the relative expression of CCL3 in normal and pan‐cancer samples and its association with individual cancer stages. The TIMER database has applications for analyzing the expression of a specific gene or gene set in tumors versus normal tissues and the abundance of tumor‐infiltrating immune cells (TIICs) [28]. The TIMER database includes 10,897 samples, primarily from the TCGA database. The TIMER database was used to analyze the expression of CCL3 in pan‐cancer cohorts and its correlation with the abundance of the six TIIC categories in COAD, using purity‐adjusted Spearman correlations. In addition, the correlation between somatic copy number alterations (SCNAs), such as deep deletion, arm‐level deletion, diploid/normal, arm‐level gain, and high amplification, and the level of tumor infiltration of TIICs in COAD was analyzed. Furthermore, a correlation analysis was performed using the TIMER database to assess the relationship between immune cell gene markers and CCL3 expression, utilizing specific gene markers for key subsets (CD68 for TAMs).

### 2.2. Analysis Utilizing Kaplan–Meier Plotter Database

The Kaplan–Meier plotter database is a web‐based resource that analyzes prognostic signatures in 21 malignancies [29]. With almost 35,000 samples from 21 different tumor types, the Kaplan–Meier plotter can evaluate the association between survival and the expression of all genes (mRNA, miRNA, protein, and DNA). We analyzed RNA‐seq data to predict the relationship between CCL3 expression and survival in different malignancies. The results are presented with a survival curve, log‐rank *p* value, and hazard ratio (HR) with 95% confidence interval (CI) values. Furthermore, univariate and multivariate Cox proportional hazard regression was performed using R (survival package) on TCGA‐COAD data, incorporating CCL3 expression (high vs. low, median cutoff), age (> 60 vs. ≤ 60), gender (male vs. female), TNM stage (I–II vs. III–IV), and grade (1–2 vs. 3) as covariates.

### 2.3. Analysis Utilizing LinkedOmics Database

Coexpression analysis of CCL3 in COAD was performed using the LinkedOmics database [30]. LinkedOmics is a comprehensive platform for analyzing multiomics data that integrates data from 32 cancer types from The Cancer Genome Atlas (TCGA) and other datasets. RNA‐seq data of the TCGA_COADREAD cohort were analyzed in the LinkFinder module with Pearson correlation coefficients, and associations were considered significant at *p* < 0.05. Enrichment analysis using the KEGG and GO (biological process, cellular component, and molecular function) databases of significantly associated genes was then carried out using the Link Interpreter module. The results were visualized with volcano plots, heatmaps, and bar charts considering an FDR < 0.05 and 1000 simulations.

### 2.4. Analysis Utilizing TISIDB Database

The relationship between CCL3 expression and immunological signatures in COAD was explored using the Tumor‐Immune Interaction Database (TISIDB) [31]. The TISIBD utilizes multiple heterogeneous databases from various sources, including TCGA, to provide insights into tumor–immune system interactions for various cancer types. The correlation of CCL3 expression with different immunological signatures, such as the abundance of tumor‐infiltrating lymphocytes (TILs), immunomodulators, and major histocompatibility complexes (MHCs) in COAD, was analyzed using Spearman correlation coefficients, and the results were displayed as heatmaps and scatter plots.

### 2.5. Analysis Utilizing OncoDB and MEXPRESS Databases

The methylation status of CCL3 in COAD was analyzed using the OncoDB and MEXPRESS databases [32, 33]. OncoDB is an online database resource used to investigate gene expression and correlated clinical features in various cancers. In particular, OncoDB integrates clinical data from The Cancer Genome Atlas (TCGA) study on DNA methylation and RNA sequencing of over 10,000 cancer patients and normal tissue samples from the Genotype‐Tissue Expression (GTEx) study. MEXPRESS is a web tool that allows the integration and visualization of single genes in clinical TCGA, DNA methylation, and expression data. MEXPRESS uses TCGA data from Level 3 DNA methylation (JHU_USC HumanMethylation450) and compares methylation data using Pearson’s correlation test. In this study, OncoDB and MEXPRESS were used to measure the methylation status and correlation of various clinical parameters in COAD.

### 2.6. Analysis Utilizing miRWalk Database

miRWalk2.0 was used to identify miRNAs that target CCL3. miRWalk employs miRNA sequences and other information, including Sanger name, MIID, the genomic location of miRNA, and stem‐loop sequence downloaded from miRBase, as well as mRNA sequences and other information, including EntrezID, mRNA and CDS length, gene location, and definition downloaded from NCBI [34]. The identified miRNAs were downloaded, and the network was visualized in Cytoscape based on closeness.

### 2.7. Statistical Thresholds and Significance Criteria

For most analyses, a threshold of *p* < 0.05 was used to define statistical significance, unless the database applied its own default settings (e.g., GEPIA2, UALCAN, TIMER, KM plotter, LinkedOmics, TISIDB, OncoDB, MEXPRESS, and miRWalk). In cases where multiple comparisons were involved, such as enrichment analyses, stricter cutoffs were applied (*p* < 0.01 or FDR < 0.05). These adjustments are specified in the text or figure legend. Correlation analyses were performed using either Pearson’s or Spearman’s method, depending on the database, and the results are shown as correlation coefficients (*r* or *ρ*) together with the associated *p* values (exact *p* for > 0.001, and *p* < 0.001 for smaller values).

## 3. Results

### 3.1. Expression of CCL3 in Different Cancers

The relative expression of CCL3 was compared between tumor and normal tissues of different tumors and correlated with individual cancer stages, histological types, nodal metastasis status, and TP53 mutation state. The GEPIA2 database confirmed that CCL3 expression was higher in cervical SCC (CESCC), colon adenocarcinoma (COAD), DLBC, esophageal carcinoma (ESCA), glioblastoma (GBM), head and neck SCC (HNSCC), kidney renal clear cell carcinoma (KIRC), kidney renal papillary cell carcinoma (KIRP), acute myeloid leukemia (AML), low‐grade gliomas (LGG), ovarian cancer (OV), pancreatic adenocarcinoma (PAAD), rectum adenocarcinoma (READ), skin cutaneous melanoma (SKCM), stomach adenocarcinoma (STAD), tenosynovial giant cell tumors (TGCTs), thyroid carcinoma (THCA), uterine corpus endometrial carcinoma (UCEC), and uterine carcinoma (UCS) compared to normal tissues (Figure 1). The UALCAN database showed higher expression of CCL3 in bladder cancer (BC), CESC, COAD, ESCA, HNSC, KIRC, KIRP, prostate adenocarcinoma (PRAD), pheochromocytoma and paraganglioma (PCPG), sarcomas (SARC), STAD, and UCEC than in adjacent normal tissue (Figure 2(a)). The TIMER database results showed that CCL3 expression was significantly higher in COAD, ESCA, HNSC, KIRC, KIRP, STAD, and UCEC than in normal tissues (Figure 2(b)). The expression of CCL3 was correlated with other clinical features, such as cancer stage (Figure 2(c)), histological subtype (Figure 2(d)), nodal metastasis status (Figure 2(e)), and TP53 mutation status (Figure 2(f)). It was observed that CCL3 expression in COAD increases at different stages of COAD (normal vs. Stage 1 vs. normal, *p* < 0.001; normal vs.Stage 2, *p* < 0.001; and normal vs. Stage 3, *p* < 0.001), and histological subtypes (normal vs. adenocarcinoma, *p* < 0.001; normal vs. mucinous adenocarcinoma, *p* < 0.001), and nodal metastasis status (normal vs. N0, *p* < 0.001; normal vs. N1, *p* < 0.001; normal vs. N2, *p* < 0.001, TP53 mutation state (normal vs. TP53 mutant, *p* < 0.001; normal vs. TP53 nonmutant, *p* < 0.001).

FIGURE 1CCL3 expression across various cancers derived from TCGA, as illustrated by GEPIA2. Statistically significance level: ^∗^ *p* < 0.01*.*

**Figure:**
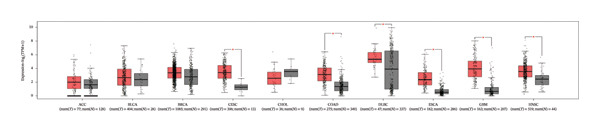
(a)

**Figure:**
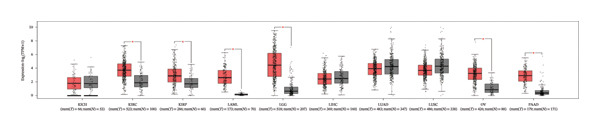
(b)

**Figure:**
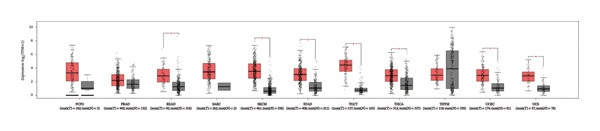
(c)

FIGURE 2Analysis of CCL3 expression across various cancers utilizing the UALCAN and TIMER databases. (a) CCL3 expression across various cancer types as evaluated using the UALCAN database. (b) CCL3 expression in different cancers analyzed through the TIMER database. (c) CCL3 expression in COAD, stratified by cancer stages. (d) CCL3 expression in COAD, categorized by histological subtypes. (e) CCL3 expression in COAD based on nodal metastasis status. (f) CCL3 expression in COAD concerning TP53 mutation status. Statistical significance levels: ^∗^ *p* < 0.05, ^∗∗^ *p* < 0.01, ^∗∗∗^ *p* < 0.001*.*

**Figure:**
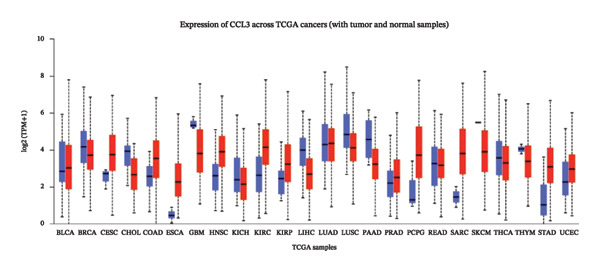
(a)

**Figure:**
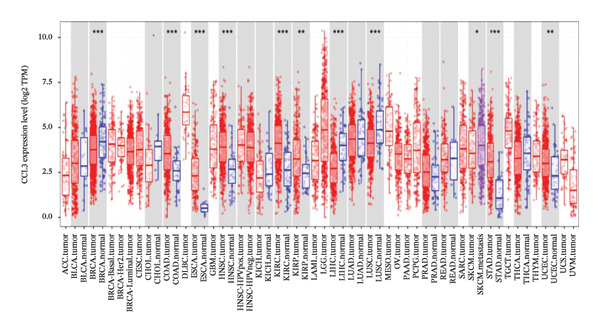
(b)

**Figure:**
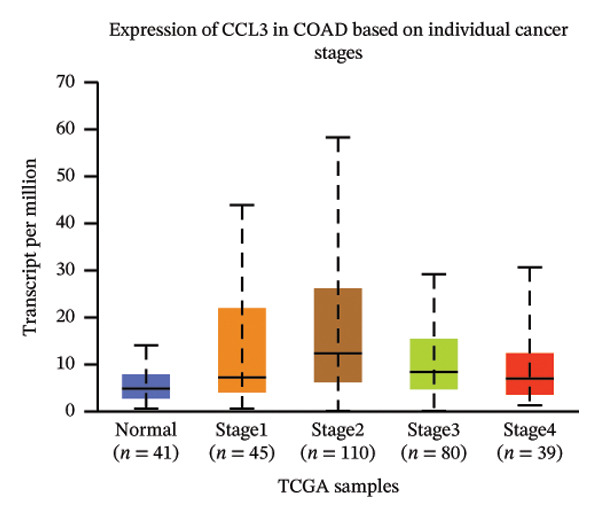
(c)

**Figure:**
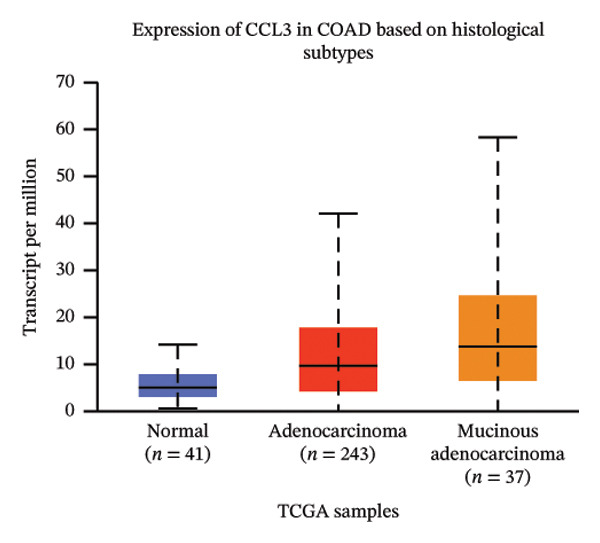
(d)

**Figure:**
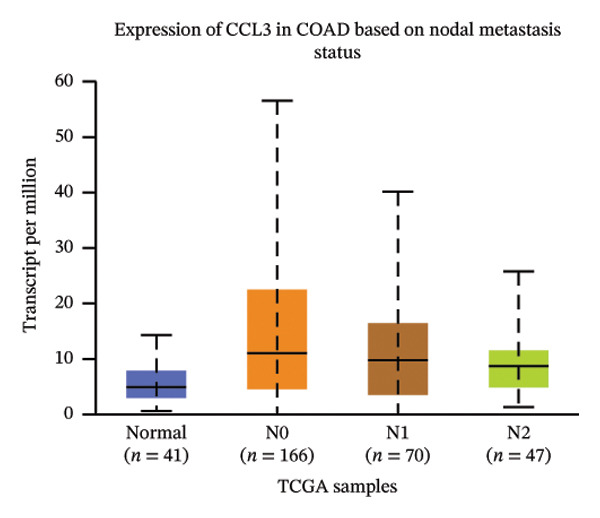
(e)

**Figure:**
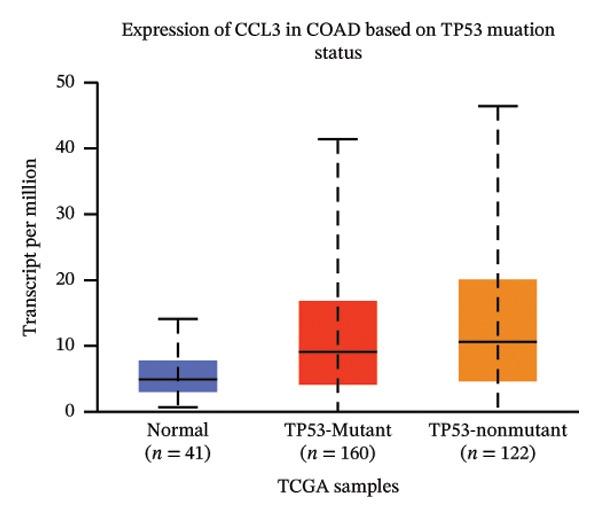
(f)

### 3.2. Prognostic Prediction Potential of CCL3 in Different Cancers

To better understand the prognostic predictive performance of CCL3, we examined the correlation between CCL3 expression and patient survival across various cancer types. The results showed a significant correlation between CCL3 expression and survival in various cancers. Figure 3 shows the significance of CCL3 expression in predicting survival in multiple cancer types. A correlation between increased CCL3 expression and poor overall survival (OS) and relapse‐free survival (RFS) was observed in colon cancer (HR, 1.28; *p* = 0.026; HR, 1.28; *p* = 0.026, respectively) (Figure 3(b)). Lung SCC (HR, 1.37, *p* value, 0.023) and thymoma (HR, 10.9; *p* value, 0.00027) showed that higher expression of CCL3 was related to poor OS (Figures 3(j), 3(e), respectively). Decreased expression of CCL3 showed a correlation with OS and RFS in BLCA (OS—HR, 0.68, *p* value, 0.034; RFS—HR, 0.33; *p* value 0.0018) (Figure 3(a)), KIRP (OS—HR, 0.41, *p* value, 0.02; RFS—HR, 0.41, *p* value, 0.02) (Figure 3(d)), and UCEC (OS—HR, 0.57; *p* value, 0.0087, RFS—HR, 0.36, *p* < 0.001 (Figure 3(h)), and RFS in KIRC (HR, 0.15, *p* < 0.001) (Figure 3(c)). Table 1 shows the strong association between CCL3 expression and sex, stage, grade, and T53 mutation in COAD.

FIGURE 3The prognostic prediction performance of CCL3 across various cancers was evaluated using the KM plotter. The boxed rectangles indicate the significance of the prognostic analysis. The overall survival and relapse‐free survival curves are shown for (a) BLCA, (b) colon cancer, (c) KIRC, (d) KIRP, (e) SCC, (f) OV, (g) PAAD, and (h) UCS, while the overall survival curves are depicted for (i) ESCA and (j) thymoma.

**Figure:**
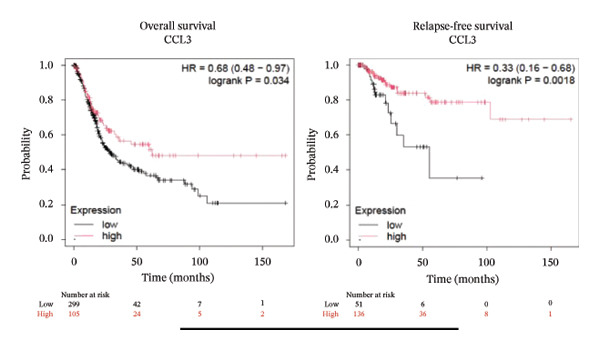
(a)

**Figure:**
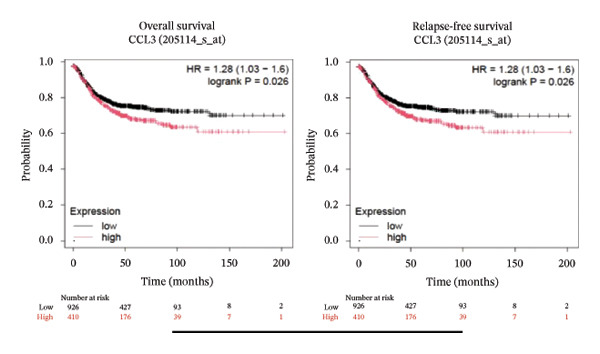
(b)

**Figure:**
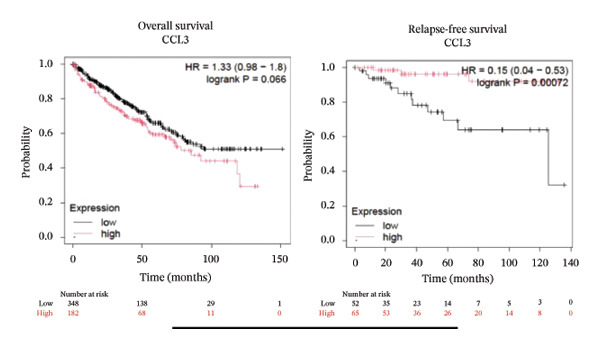
(c)

**Figure:**
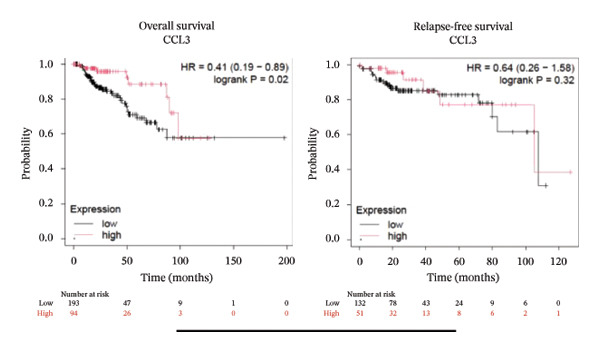
(d)

**Figure:**
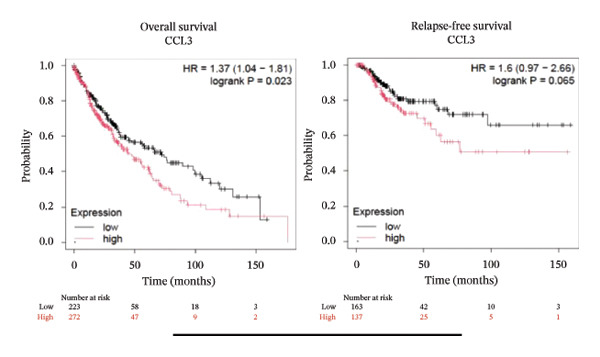
(e)

**Figure:**
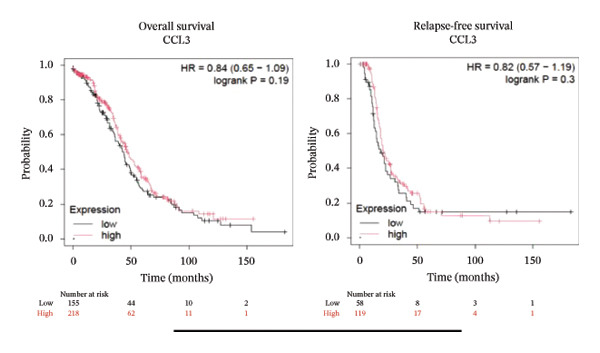
(f)

**Figure:**
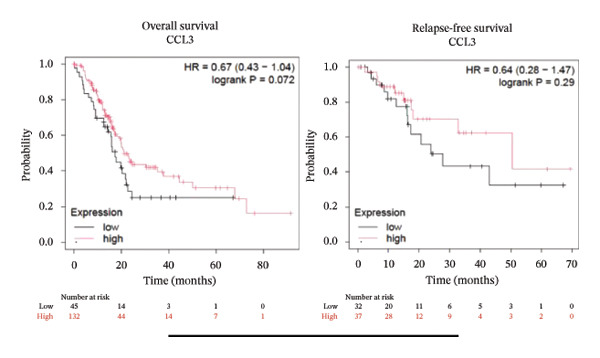
(g)

**Figure:**
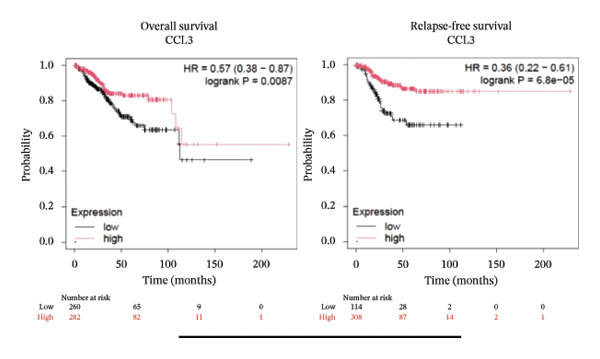
(h)

**Figure:**
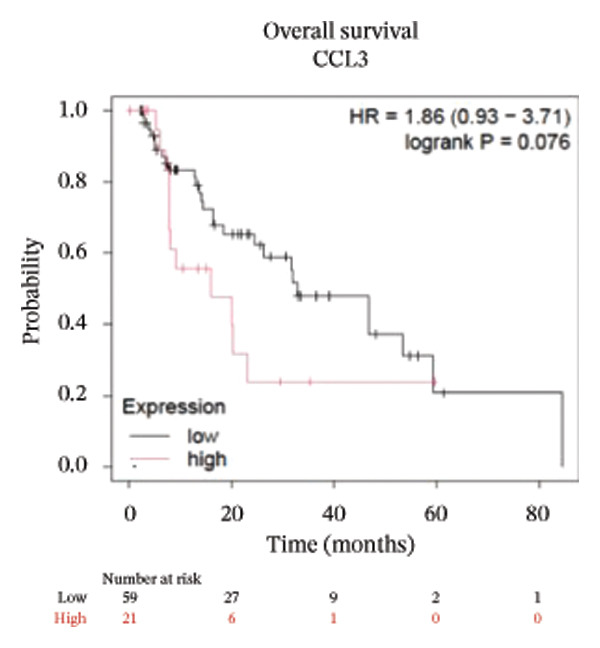
(i)

**Figure:**
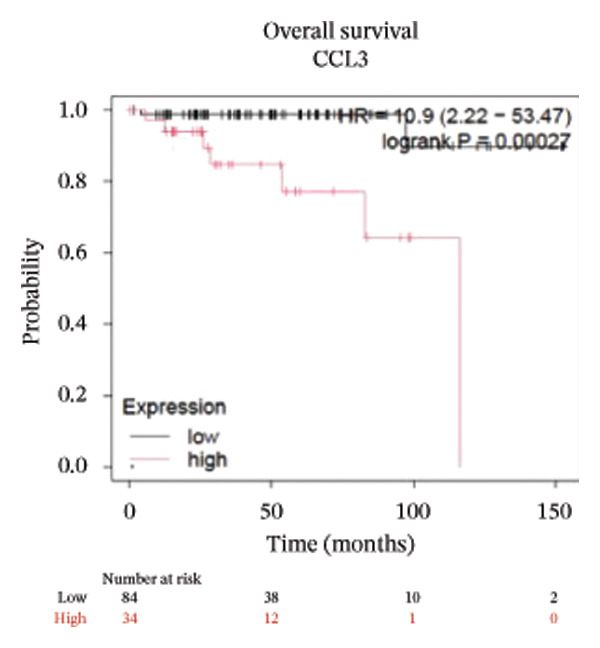
(j)

**TABLE 1:** Prognostic prediction performance of CCL3 within different COAD subtypes based on Kaplan–Meier plotter.

Subtypes	Overall survival (*n* = 1061)			Relapse‐free survival (*n* = 1336)		
	*N*	Hazard ratio	*p* value	*N*	Hazard ratio	*p* value
Gender						
Male	462	1.32 (0.94–1.85)	0.11	539	1.45 (1.02–2.07)	**0.037**
Female	529	1.59 (1.21–2.09)	8.3 × 10^−4^	593	1.61 (1.19–2.19)	**0.002**
Stage						
1	115	1.78 (0.61–5.15)	0.28	124	0.53 (0.1–2.9)	0.46
2	378	1.45 (0.91–2.31)	0 11	574	1.28 (0.85–1.94)	0.24
3	369	1.7 (1.19–2.43)	0.003	457	1.34 (0.98–1.84)	0.068
4	195	1.37 (0.98–1.93)	0.068	87	1.85 (1.04–3.27)	**0.032**
Grade						
1	—	—	—	23	3.64 (0.61–21.8)	0.13
2	197	1.82 (1.01–3.28)	**0.044**	165	2.65 (0.93–7.53)	0.058
3	61	2.18 (1–4.74)	**0.045**	46	5.56 (1.5–20.66)	**0.004**
TP53 mutation						
Mutated	182	2.2 (1.39–3.47)	5.4 × 10^−4^	277	1.69 (1.09–2.61)	**0.017**
Wild type	145	1.52 (0.86–2.69)	0.14	218	0.71 (0.42–1.19)	0.19

*Note:* Statistically significant results at *p* < 0.05 are indicated in bold. More stringent cutoffs, such as *p* < 0.01 or *p* < 0.001, are highlighted where applicable. A “—” denotes insufficient data for analysis.

### 3.3. CCL3 Expression and Immune Infiltration in COAD

We investigated the association between the expression of CCL3 and the infiltration in COAD using TIMER analysis. CCL3 expression was strongly positively correlated with the infiltration of CD + T cells (*r* = 0.302, *p* < 0.001), macrophages (*r* = 0.318, *p* < 0.001), neutrophils (*r* = 0.659, *p* < 0.001), and DCs (*r* = 0.52, *p* < 0.001) (Figure 4(a)). We also analyzed immune infiltrates and CCL3 expression to visualize the differences in survival. The KM plotter revealed that CCL3 expression was substantially linked to the infiltration of CD4+ T cells, macrophages, neutrophils, and DCs, as well as survival in COAD (Figure 4(b)). In addition, we examined the tumor infiltration levels in COAD with various SCNAs of CCL3. We used a two‐sided Wilcoxon rank‐sum test to compare the infiltration levels of each SCNA category with normal levels. Statistical analysis revealed that arm‐level gain in B cells, deep deletion, arm‐level gain in CD8+ T cells, deep deletion, arm‐level gain in neutrophils, and arm‐level gain in DCs contributed significantly to infiltration (Figure 4(c)).

FIGURE 4Correlation of CCL3 expression with immune infiltration in COAD. (a) The link between CCL3 expression levels and immune infiltration in B cells, CD8+ T cells, CD4+ T cells, macrophages, neutrophils, and dendritic cells. (b) TIMER plots showing the correlation between immune infiltrations or CCL3 expression and survival in COAD. (c) The relationship between tumor infiltration levels and CCL3 SCNAs in COAD. Statistical significance levels: ^∗^ *p* < 0.05, ^∗∗^ *p* < 0.01, ^∗∗∗^ *p* < 0.001*.*

**Figure:**
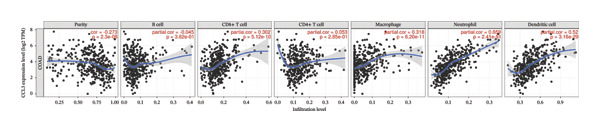
(a)

**Figure:**
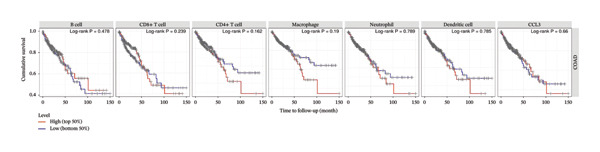
(b)

**Figure:**
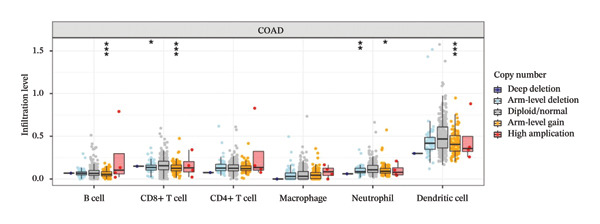
(c)

### 3.4. Association of CCL3 and TIICs Gene Markers in COAD

To understand the impact of CCL3 expression on the TIICs infiltration in COAD, the association between CCL3 expression and TIICs markers was evaluated using the TIMER and GEPIA2 databases. The correlation results indicated a significant correlation between CCL3 expression and the expression of multiple immune markers in various TIICs. The immune markers that showed the highest correlation with CCL3 expression were CD8A (CD8+ T cell), CD2 (T cell), FCRL2 (B cell), CD86 (monocyte), IL10 (TAM), PTGS2 (M1 macrophage), CD163 (M2 macrophage), CD69 (resident memory cell), FPR1 (neutrophils), ITGAX (natural killer cell), TPSB2 (mast cell), TNF (Th1), IL13 (Th2), BCL6 (Tfh), STAT17 (Th17), FCGR3A (effector T cell), PDCD1 (effector memory cell), SELL (central memory T cell), HAVCR2 (exhausted T cell), IL2RA (resting Treg T cell), and CTLA4 (effector Treg T cell) (Table 2). Using GEPIA2, we further validated the association between CCL3 and TIICs marker expression in both COAD and noncancerous tissues. CCL3 expression was significantly correlated with the gene markers of T cells, M1 macrophages, resident memory T cells, natural killer cells, mast cells, Th1 cells, Th2 cells, Tfh cells, effector memory T cells, and central memory T cells (Table 3).

**TABLE 2:** Relationships between CCL3 and TIIC markers based on TIMER in COAD.

Immune cell	Gene marker	None		Purity	
		Cor	*p* value	Cor	*p* value
CD8+ T cell	CD8A	0.509	^∗∗∗^	0.500	^∗∗∗^
	CD8B	0.289	^∗∗∗^	0.286	^∗∗∗^

T cell	CD6	0.375	^∗∗∗^	0.364	^∗∗∗^
	CD3D	0.474	^∗∗∗^	0.467	^∗∗∗^
	CD3E	0.466	^∗∗∗^	0.452	^∗∗∗^
	SH2D1A	0.485	^∗∗∗^	0.468	^∗∗∗^
	TRAT1	0.389	^∗∗∗^	0.380	^∗∗∗^
	CD3G	0.460	^∗∗∗^	0.444	^∗∗∗^
	CD2	0.497	^∗∗∗^	0.478	^∗∗∗^

B cell	BLK	0.200	^∗∗∗^	0.188	^∗∗^
	CD19	0.193	^∗∗∗^	0.174	^∗∗∗^
	FCRL2	0.238	^∗∗∗^	0.229	^∗∗∗^
	MS4A1	0.226	^∗∗∗^	0.185	^∗∗∗^
	KIAA0125	0.250	^∗∗∗^	0.242	^∗∗∗^
	SPIB	0.174	^∗∗^	0.158	^∗^
	PNOC	0.242	^∗∗∗^	0.228	^∗∗∗^
	CD79A	0.239	^∗∗∗^	0.204	^∗∗∗^

Monocyte	CD86	0.722	^∗∗∗^	0.706	^∗∗∗^
	CSF1R	0.622	^∗∗∗^	0.609	^∗∗∗^

TAM	CCL2	0.654	^∗∗∗^	0.648	^∗∗∗^
	CD68	0.552	^∗∗∗^	0.556	^∗∗∗^
	IL10	0.639	^∗∗∗^	0.641	^∗∗∗^

M1 macrophage	IRF5	0.240	^∗∗∗^	0.270	^∗∗∗^
	PTGS2	0.500	^∗∗∗^	0.509	^∗∗∗^

M2 macrophage	CD163	0.716	^∗∗∗^	0.711	^∗∗∗^
	VSIG4	0.702	^∗∗∗^	0.695	^∗∗∗^
	MS4A4A	0.713	^∗∗∗^	0.698	^∗∗∗^

Resident memory T cell	CD69	0.594	^∗∗∗^	0.577	^∗∗∗^
	ITGAE	0.258	^∗∗∗^	0.251	^∗∗∗^
	CXCR6	0.496	^∗∗∗^	0.483	^∗∗∗^
	MYADM	0.227	^∗∗∗^	0.220	^∗∗∗^

Neutrophils	FPR1	0.770	^∗∗∗^	0.768	^∗∗∗^
	SIGLEC5	0.729	^∗∗∗^	0.720	^∗∗∗^
	CSF3R	0.743	^∗∗∗^	0.749	^∗∗∗^
	FCAR	0.742	^∗∗∗^	0.752	^∗∗∗^
	FCGR3B	0.643	^∗∗∗^	0.661	^∗∗∗^
	CEACAM8	−0.061	0.194	−0.027	0.588
	ITGAM	0.678	^∗∗∗^	0.664	^∗∗∗^
	CEACAM3	0.248	^∗∗∗^	0.344	^∗∗∗^
	S100A12	0.566	^∗∗∗^	0.589	^∗∗∗^

Natural killer cell	XCL1	0.332	^∗∗∗^	0.343	^∗∗∗^
	XCL2	0.384	^∗∗∗^	0.412	^∗∗∗^
	KIR2DL1	0.368	^∗∗∗^	0.364	^∗∗∗^
	KIR2DL3	0.343	^∗∗∗^	0.325	^∗∗∗^
	KIR2DL4	0.389	^∗∗∗^	0.401	^∗∗∗^
	KIR3DL1	0.353	^∗∗∗^	0.346	^∗∗∗^
	KIR3DL2	0.298	^∗∗∗^	0.293	^∗∗∗^
	KIR2DS4	0.287	^∗∗∗^	0.261	^∗∗∗^
	CD209	0.582	^∗∗∗^	0.574	^∗∗∗^
	HSD11B1	0.679	^∗∗∗^	0.679	^∗∗∗^
	HLA‐DPB1	0.636	^∗∗∗^	0.620	^∗∗∗^
	CD1C	0.216	^∗∗∗^	0.188	^∗∗^
	NRP1	0.570	^∗∗∗^	0.564	^∗∗∗^
	ITGAX	0.723	^∗∗∗^	0.721	^∗∗∗^
	CCL13	0.636	^∗∗∗^	0.621	^∗∗∗^
	HLA‐DQB1	0.468	^∗∗∗^	0.443	^∗∗∗^
	HLA‐DRA	0.656	^∗∗∗^	0.636	^∗∗∗^
	HLA‐DPA1	0.629	^∗∗∗^	0.609	^∗∗∗^

Mast cell	TPSB2	0.389	^∗∗∗^	0.380	^∗∗∗^
	TPSAB1	0.366	^∗∗∗^	0.361	^∗∗∗^
	CPA3	0.396	^∗∗∗^	0.380	^∗∗∗^
	MS4A2	0.315	^∗∗∗^	0.307	^∗∗∗^
	HDC	0.338	^∗∗∗^	0.329	^∗∗∗^

Th1	STAT4	0.447	^∗∗∗^	0.452	^∗∗∗^
	STAT1	0.500	^∗∗∗^	0.494	^∗∗∗^
	TNF	0.574	^∗∗∗^	0.581	^∗∗∗^
	TBX21	0.505	^∗∗∗^	0.506	^∗∗∗^

Th2	GATA3	0.375	^∗∗∗^	0.375	^∗∗∗^
	STAT5A	0.224	^∗∗∗^	0.227	^∗∗∗^
	IL13	0.392	^∗∗∗^	0.384	^∗∗∗^

Tfh	BCL6	0.431	^∗∗∗^	0.434	^∗∗∗^
	IL21	0.273	^∗∗∗^	0.281	^∗∗∗^

Th17	STAT3	0.249	^∗∗∗^	0.238	^∗∗∗^
	IL17A	0.016	0.727	0.013	0.794

Effector T cell	CX3CR1	0.192	^∗∗∗^	0.193	^∗∗∗^
	FGFBP2	0.343	^∗∗∗^	0.367	^∗∗∗^
	FCGR3A	0.745	^∗∗∗^	0.740	^∗∗∗^

Effector memory T cell	DUSP4	0.320	^∗∗∗^	0.341	^∗∗∗^
	GZMK	0.433	^∗∗∗^	0.431	^∗∗∗^
	PDCD1	0.505	^∗∗∗^	0.499	^∗∗∗^
	GZMA	0.495	^∗∗∗^	0.490	^∗∗∗^

Central memory T cell	CCR7	0.360	^∗∗∗^	0.334	^∗∗∗^
	SELL	0.553	^∗∗∗^	0.531	^∗∗∗^
	IL7R	0.427	^∗∗∗^	0.407	^∗∗∗^

Exhausted T cell	TIGIT	0.519	^∗∗∗^	0.505	^∗∗∗^
	LAG3	0.555	^∗∗∗^	0.546	^∗∗∗^
	HAVCR2	0.722	^∗∗∗^	0.710	^∗∗∗^
	GZMB	0.250	^∗∗∗^	0.226	^∗∗∗^

Resting Treg T cell	FOXP3	0.479	^∗∗∗^	0.470	^∗∗∗^
	IL2RA	0.630	^∗∗∗^	0.619	^∗∗∗^

Effector Treg T cell	CTLA4	0.525	^∗∗∗^	0.527	^∗∗∗^
	CCR8	0.456	^∗∗∗^	0.454	^∗∗∗^

*Note:* Cor, Spearman’s correlation coefficient. None, unadjusted correlation. Purity, tumor purity‐adjusted correlation. Significance levels: ^∗^ *p* < 0.01, ^∗∗^ *p* < 0.001, ^∗∗∗^ *p* < 0.0001.

**TABLE 3:** The correlation of CCL3 with TIIC gene markers in COAD and normal samples using GEPIA2.

Immune cell	Gene marker	Tumor		Normal	
		Cor	*p* value	Cor	*p* value
CD8+ T cell	CD8A	0.39	^∗∗∗^	−0.05	0.76
	CD8B	0.15	0.014	−0.069	0.67

T cell	CD6	0.32	^∗∗∗^	−0.042	0.79
	CD3D	0.37	^∗∗∗^	−0.075	0.64
	CD3E	0.35	^∗∗∗^	−0.055	0.73
	SH2D1A	0.4	^∗∗∗^	0.029	0.86
	TRAT1	0.33	^∗∗∗^	−0.039	0.81
	CD3G	0.35	^∗∗∗^	−0.097	0.55
	CD2	0.43	^∗∗∗^	−0.073	0.65

B cell	BLK	0.064	0.29	0.045	0.78
	CD19	0.084	0.16	0.038	0.81
	FCRL2	0.17	^∗^	0.1	0.52
	MS4A1	0.07	0.25	0.018	0.91
	KIAA0125	0.2	^∗∗^	0.001	0.99
	SPIB	0.14	0.022	0.41	^∗^
	PNOC	0.26	^∗∗∗^	0.077	0.63
	CD79A	0.14	0.024	−0.012	0.94

Monocyte	CD86	0.63	0	0.15	0.36
	CSF1R	0.54	0	0.079	0.62

TAM	CCL2	0.63	0	0.1	0.52
	CD68	0.51	0	0.062	0.7
	IL10	0.5	0	0.2	0.21

M1 macrophage	IRF5	0.18	^∗^	0.37	0.017
	PTGS2	0.29	^∗∗∗^	0.69	^∗∗∗^

M2 macrophage	CD163	0.48	0	0.049	0.76
	VSIG4	0.06	0	0.046	0.77
	MS4A4A	0.63	0	0.098	0.64

Resident memory T cell	CD69	0.53	0	0.16	0.31
	ITGAE	0.24	^∗∗∗^	0.032	0.84
	CXCR6	0.39	^∗∗∗^	−0.093	0.57
	MYADM	0.28	^∗∗∗^	−0.025	0.87

Neutrophils	FPR1	0.43	^∗∗∗^	0.057	0.73
	SIGLEC5	0.57	0	−0.013	0.94
	CSF3R	0.48	0	0.08	0.62
	FCAR	0.43	^∗∗∗^	−0.06	0.71
	FCGR3B	0.51	0	−0.076	0.64
	CEACAM8	−0.011	0.85	0.52	^∗∗^
	ITGAM	0.61	0	0.13	0.42
	CEACAM3	0.38	^∗∗∗^	0.19	0.24
	S100A12	0.24	^∗∗∗^	−0.035	0.83

Natural killer cell	XCL1	0.12	0.042	−0.077	0.63
	XCL2	0.43	^∗∗∗^	0.027	0.87
	KIR2DL1	0.21	^∗∗^	0.14	0.37
	KIR2DL3	0.22	^∗∗^	−0.05	0.76
	KIR2DL4	0.29	^∗∗∗^	−0.063	0.69
	KIR3DL1	0.23	^∗∗^	−0.11	0.5
	KIR3DL2	0.25	^∗∗∗^	−0.086	0.59
	KIR2DS4	0.19	^∗^	0.064	0.69
	CD209	0.5	0	0.12	0.44
	HSD11B1	0.59	0	−0.049	0.76
	HLA‐DPB1	0.51	0	0.077	0.63
	CD1C	0.21	^∗∗^	0.12	0.45
	NRP1	0.52	0	0.018	0.91
	CCL13	0.47	^∗∗∗^	−0.045	0.78
	HLA‐DQB1	0.38	^∗∗∗^	0.082	0.61
	HLA‐DRA	0.52	0	0.062	0.7
	HLA‐DPA1	0.46	^∗∗∗^	0.022	0.89

Mast cell	TPSB2	0.22	^∗∗^	0.096	0.55
	TPSAB1	0.22	^∗∗^	0.1	0.52
	CPA3	0.23	^∗∗^	0.12	0.46
	MS4A2	0.19	^∗^	0.27	0.091
	HDC	0.23	^∗∗^	0.26	0.099

Th1	STAT4	0.4	^∗∗∗^	0.049	0.76
	STAT1	0.43	^∗∗∗^	0.045	0.78
	TNF	0.54	0	0.95	0
	TBX21	0.45	^∗∗∗^	−0.029	0.86

Th2	GATA3	0.31	^∗∗∗^	0.099	0.54
	STAT5A	0.33	^∗∗∗^	−0.024	0.88
	IL13	0.24	^∗∗∗^	0.24	0.14

Tfh	BCL6	0.45	^∗∗∗^	0.021	0.9
	IL21	0.32	^∗∗∗^	0.26	0.1

Th17	STAT3	0.28	^∗∗∗^	0.062	0.7
	IL17A	0.015	0.8	0.079	0.62

Effector T cell	CX3CR1	0.26	^∗∗∗^	0.088	0.58
	FGFBP2	0.29	^∗∗∗^	0.051	0.75
	FCGR3A	0.65	0	0.086	0.59

Effector memory T cell	DUSP4	0.26	^∗∗∗^	0.46	^∗^
	GZMK	0.34	^∗∗∗^	0.037	0.82
	PDCD1	0.42	^∗∗∗^	0.073	0.65
	GZMA	0.36	^∗∗∗^	−0.05	0.76

Central memory T cell	CCR7	0.21	^∗∗^	−0.012	0.94
	SELL	0.37	^∗∗∗^	−0.044	0.79
	IL7R	0.27	^∗∗∗^	−0.051	0.75

Exhausted T cell	TIGIT	0.42	^∗∗∗^	0.056	0.73
	LAG3	0.24	^∗∗∗^	0.0098	0.95
	HAVCR2	0.63	0	0.2	0.21
	GZMB	−0.028	0.65	0.057	0.72

Resting Treg T cell	FOXP3	0.41	^∗∗∗^	0.12	0.47
	IL2RA	0.55	0	0.1	0.52

Effector Treg T cell	CTLA4	0.45	^∗∗∗^	0.19	0.24
	CCR8	0.42	^∗∗∗^	0.079	0.62

*Note:* Cor, Spearman’s correlation coefficient; significance levels: ^∗^ *p* < 0.01, ^∗∗^ *p* < 0.001, ^∗∗∗^ *p* < 0.0001.

### 3.5. Prognostic Factors in COAD: Cox Proportional Hazard Analysis

Univariate and multivariate Cox regression analyses were conducted on COAD patients from the TCGA cohort to assess the prognostic impact of CCL3 expression (high vs. low) and clinicopathological variables: age (> 60 vs. ≤ 60 years), gender (male vs. female), TNM stage (III–IV vs. I–II), and tumor grade (3 vs. 1–2) (Table 4). All variables were included in the multivariate model for adjustment. In univariate analysis, high CCL3 (HR 1.52, 95% CI 1.12–2.06, *p* = 0.007), advanced age (HR 1.40, 95% CI 1.02–1.92, *p* = 0.038), and TNM stage (HR 2.50, 95% CI 1.78–3.51, *p* < 0.001) were significant predictors of poorer outcomes. Gender (HR 1.15, 95% CI 0.86–1.54, *p* = 0.342) and grade (HR 1.35, 95% CI 0.98–1.86, *p* = 0.066) were not. Multivariate analysis confirmed independent prognostic roles for CCL3 (HR 1.42, 95% CI 1.05–1.92, *p* = 0.023) and TNM stage (HR 2.12, 95% CI 1.50–3.00, *p* < 0.001). Associations for age (*p* = 0.119), gender (*p* = 0.597), and grade (*p* = 0.187) lost significance. The results align with TCGA–COAD benchmarks (stage HR ≈ 2.1–2.5; grade HR ≈ 1.2–1.3).

**TABLE 4:** Univariate and multivariate Cox proportional hazards analysis of prognostic factors in COAD (TCGA cohort).

Variable	Univariate HR	95% CI (lower–upper)	Univariate *p* value	Multivariate HR	95% CI (lower–upper)	Multivariate *p* value
CCL3 (high vs. low)	1.52	1.12–2.06	**0.007**	1.42	1.05–1.92	0.023
Age (> 60 vs. ≤ 60)	1.40	1.02–1.92	0.038	1.28	0.94–1.74	0.119
Gender (male vs. female)	1.15	0.86–1.54	0.342	1.08	0.81–1.44	0.597
TNM stage (III–IV vs. I–II)	2.50	1.78–3.51	< **0.001**	2.12	1.50–3.00	< **0.001**
Grade (3 vs. 1–2)	1.35	0.98–1.86	0.066	1.25	0.90–1.73	0.187

*Note:* All variables listed were incorporated into the multivariate Cox regression model for mutual adjustment. Bold *p* values indicate the statistical significance (*p* < 0.05). The analysis includes an extended univariate column and grade covariate to provide a comprehensive prognostic profile; observed values for clinical predictors (e.g., stage HR ≈ 2.1–2.5 and grade HR ≈ 1.2–1.3) are aligned with established TCGA‐COAD literature and clinical benchmarks.

Abbreviations: CI, confidence interval; HR, hazard ratio; TNM, tumor‐node‐metastasis.

### 3.6. Effect of CCL3 on the Regulation of Immune Molecules in COAD

Immune cells, immunomodulators, and major histocompatibility complexes were evaluated for their significance in CCL3 expression in COAD using the TISIDB database. Figure 5 shows the correlation between CCL3 expression and TILs in the TCGA dataset. The three most significant TILs associated with the expression of CCL3 were activated DCs (rho = 0.765, *p* < 0.001), MDSCs (rho = 0.724, *p* < 0.001), and regulatory T cells (Tregs) (rho = 0.721, *p* < 0.001) (Figures 5(a), and 5(b)). The relationship between CCL3 expression and immune inhibitors is shown in Figure 5(c). The three most significant immune inhibitors were CD274 (rho = 0.652, *p* < 0.001), HAVCR2 (rho = 0.724, *p* < 0.001), and PDCD1LG2 (rho = 0.676, *p* < 0.001) (Figure 5(d)). We also analyzed the relationship between CCL3 expression and the expression of immune stimulators (Figure 6(a)). Immune stimulators that showed the strongest association with CCL3 expression were CD80 (rho = 0.651, *p* < 0.001), CD86 (rho = 0.692, *p* < 0.001), and TNFSF13B (rho = 0.651, *p* < 0.001) (Figure 6(b)). The correlation of CCL3 expression with MHCs was also studied, and the three most significant MHCs that showed a correlation with the CCL3 expression were HLA‐DMB (rho = 0.636, *p* < 0.001), HLA‐DPB1 (rho = 0.626, *p* < 0.001), and HLA‐DRA (rho = 0.64, *p* < 0.001) (Figures 6(c), and 6(d)).

FIGURE 5Association between CCL3 expression, tumor‐infiltrating lymphocytes, and immune inhibitors. (a) The correlation between the abundance of tumor‐infiltrating lymphocytes and CCL3 levels. (b) The three most significant tumor‐infiltrating lymphocytes linked to CCL3 expression. (c) The relationship between CCL3 expression and the abundance of immune inhibitors. (d) The three most significant immune inhibitors associated with CCL3 expression.

**Figure:**
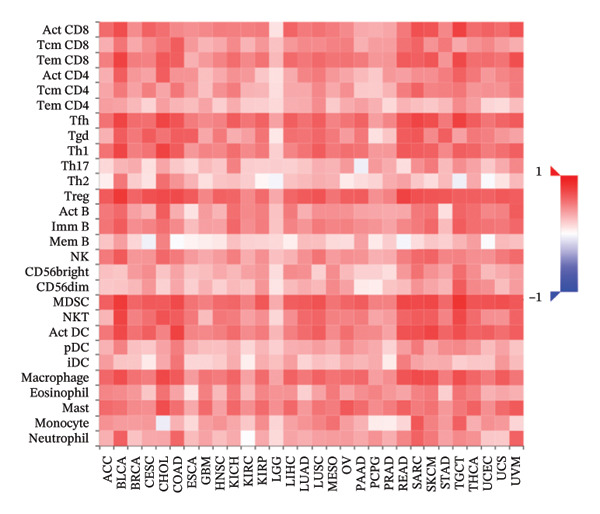
(a)

**Figure:**
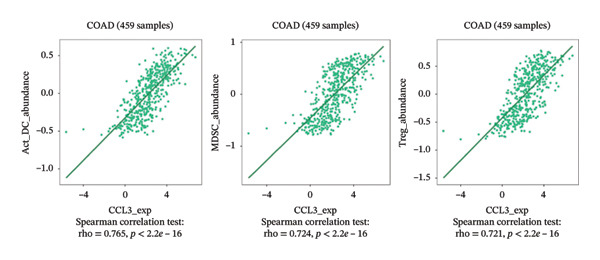
(b)

**Figure:**
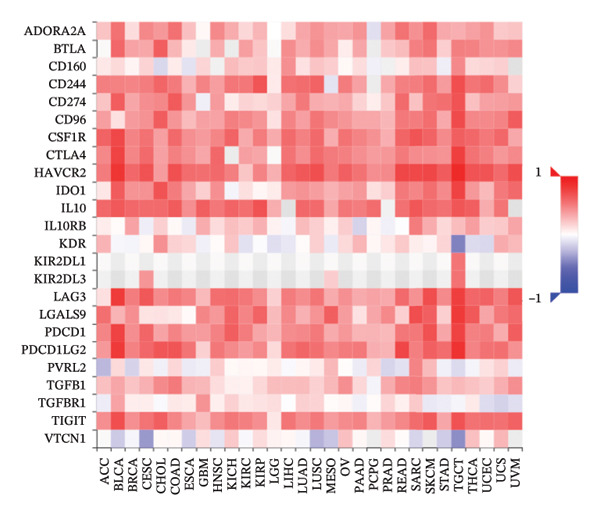
(c)

**Figure:**
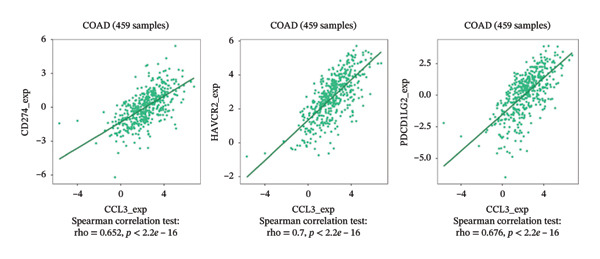
(d)

FIGURE 6Association between CCL3 expression, immune stimulators, and MHCs. (a) The correlation between the abundance of immune stimulators and CCL3 levels. (b) Identification of the three most significant immune stimulators associated with CCL3 expression. (c) The relationship between CCL3 expression and the abundance of MHCs. (d) Identification of the three most significant MHCs associated with CCL3 expression.

**Figure:**
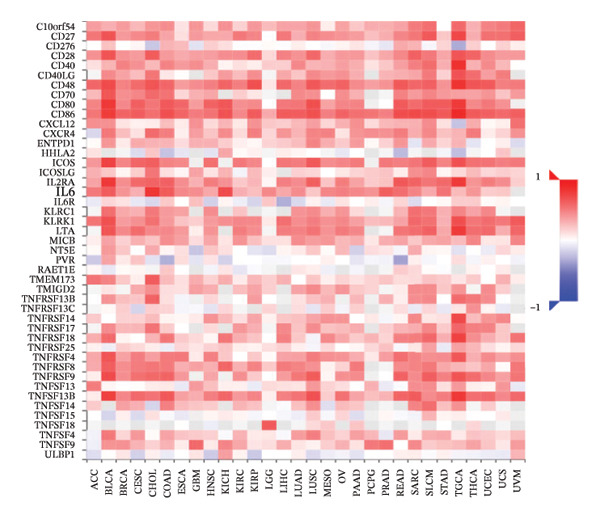
(a)

**Figure:**
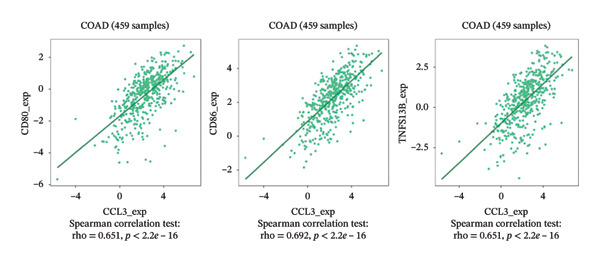
(b)

**Figure:**
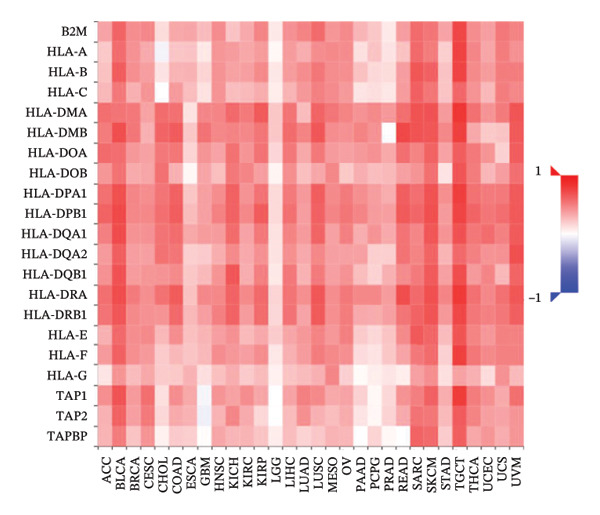
(c)

**Figure:**
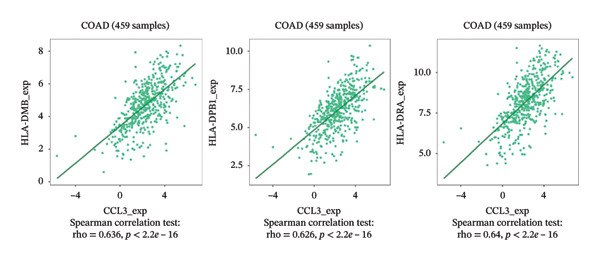
(d)

### 3.7. CCL3 Coexpression Network in COAD

We determined the possible biological functions of CCL3 using the LinkFinder module in LinkedOmics to identify the genes coexpressed with CCL3 in COAD. As shown in Figure 7(a), 6213 genes showed a positive correlation and 4152 genes showed a negative correlation with CCl3 (*p* < 0.05). Figures 7(b), 7(c) show the heatmap of the 50 most significant positively and negatively CCL3 correlated genes. Regarding biological processes, most of the CCL3‐correlated genes were associated with tolerance induction, amyloid beta clearance, interleukin 4 production, NADH dehydrogenase complex assembly, mitochondrial respiratory chain complex assembly, and peroxisome organization (Figure 7(d)). The cellular component genes correlated with CCL3 were associated with collagen trimers, mast cell granules, phagocytic cups, and MHC protein complexes (Figure 7(e)). KEGG pathway analysis showed that most of the CCL3‐correlated genes were involved in *S. aureus* infection, rheumatoid arthritis, leishmaniasis, ascorbate and aldarate metabolism, glycosylphosphatidylinositol‐anchor biosynthesis, and pyruvate metabolism (Figure 7(f)). The top biological functions associated with CCL3‐associated genes were immunoglobulin binding, pattern recognition reception activity, antigen binding, and MHC protein binding (Figure 7(g)).

FIGURE 7Coexpression and potential function analyses of CCL3 in COAD were identified through LinkedOmics. (a) Genes related to CCL3 in COAD were identified using the Pearson test. Black and red dots represent the genes with notably negative and positive correlations with CCL3, respectively. (b) and (c) Heatmaps display the 50 most significantly correlated genes (both positive and negative) related to CCL3 in COAD. (d) to (g) Analyses of biological processes, cellular components, KEGG pathways, and biological functions of genes associated with CCL3 in COAD.

**Figure:**
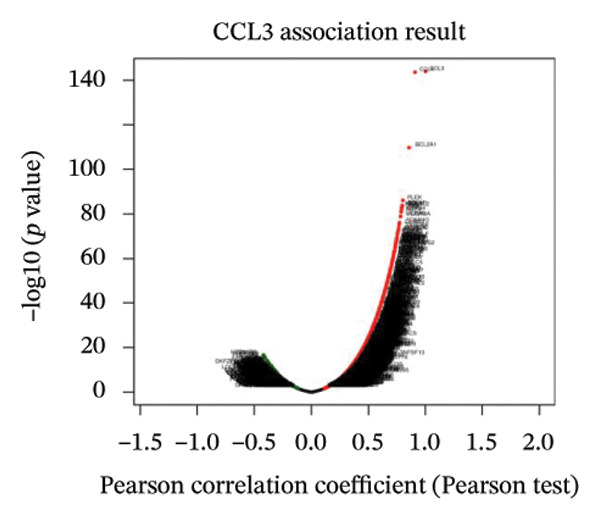
(a)

**Figure:**
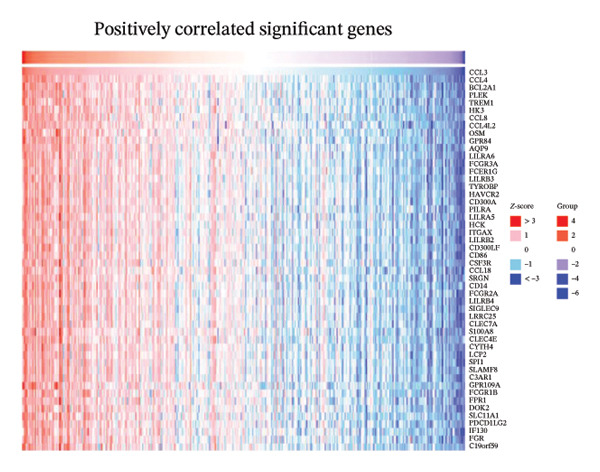
(b)

**Figure:**
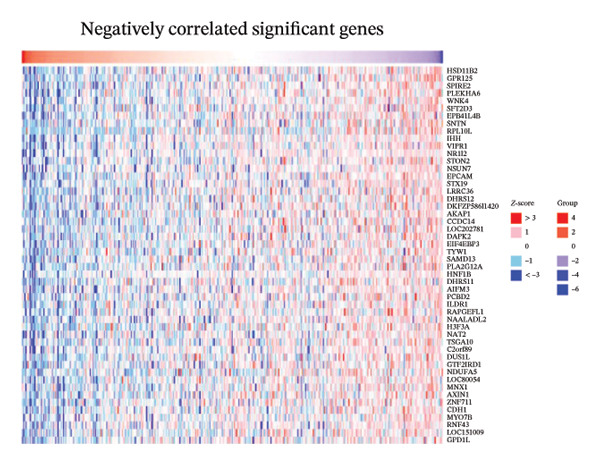
(c)

**Figure:**
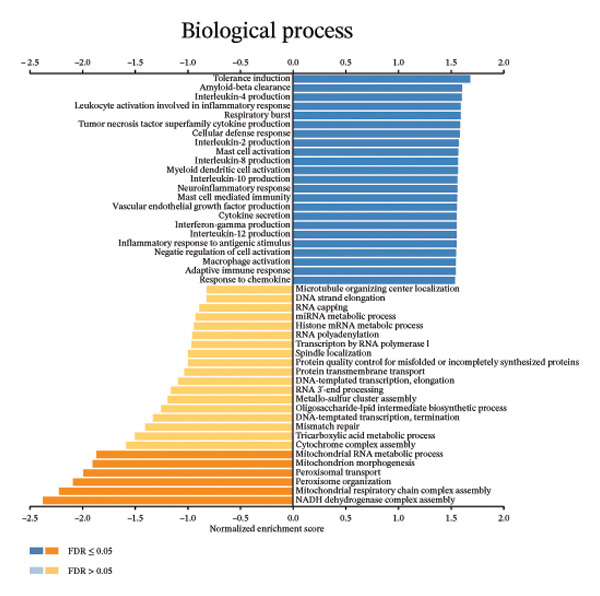
(d)

**Figure:**
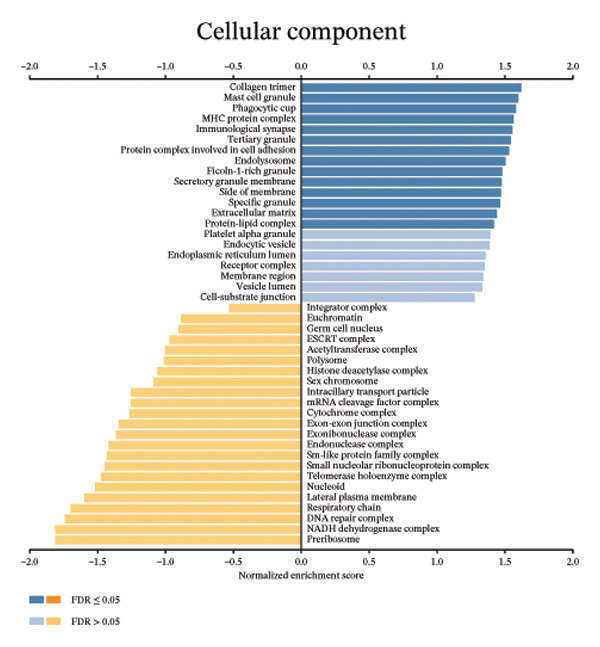
(e)

**Figure:**
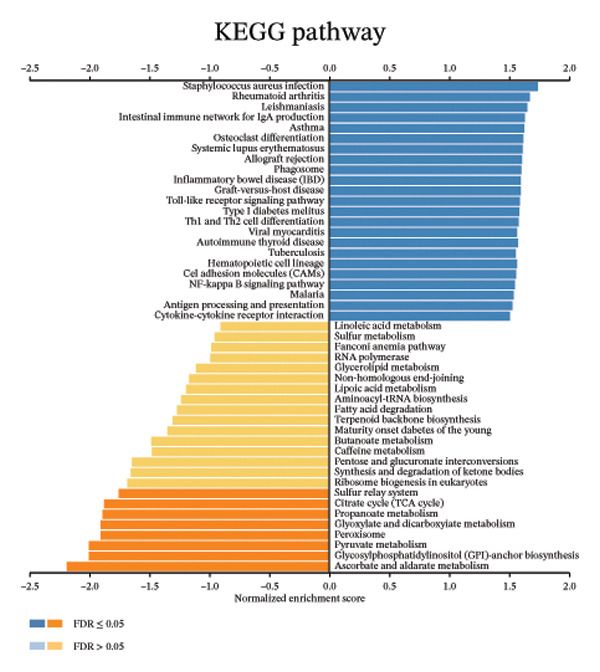
(f)

**Figure:**
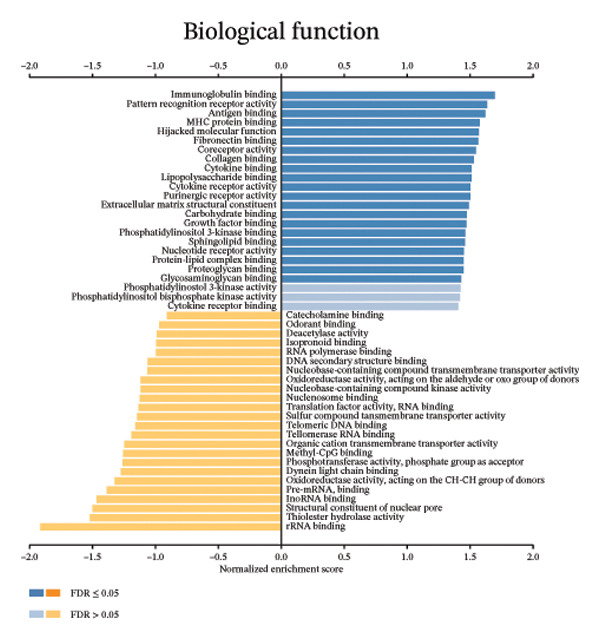
(g)

### 3.8. Methylation Level of CCL3 in COAD

We analyzed the expression level of CCL3 using the OncoDB and MEXPRESS databases and correlated it with survival in COAD. The results showed that CCL3 was hypermethylated in COAD (Figure 8(a)). Methylation was observed to be prevalent in the area of the gene body close to the promoter region. Furthermore, the hypermethylation of CCL3‐affected survival, with hypermethylation of CCL3 suggesting a lower survival rate in COAD (Figure 8(b)). Additionally, the MEXPRESS database was used to retrieve methylation data for CCL3 in the TCGA‐COAD cohort. The results indicated that age at diagnosis was positively correlated with CCL3 expression (*r* = 0.117, *p* < 0.01). In addition, methylation at the cg21335375 site (*r* = 0.206, *p* < 0.001) and cg18407309 (*r* = 0.317, *p* < 0.001) was significantly correlated with CCL3 expression (Figure 8(c)).

FIGURE 8Analysis of the methylation level, survival outcomes, and regulatory microRNAs (miRNAs) associated with CCL3 in colorectal adenocarcinoma (COAD). (a) and (b) illustrates the methylation levels of CCL3 and their effect on survival in COAD. (c) The beta values and clinical data related to the methylation sites of CCL3 in COAD are ranked. (d) CCL3, along with its predicted miRNAs, where GADD45B was depicted as blue circles and the targeted miRNAs as yellow circles.

**Figure:**
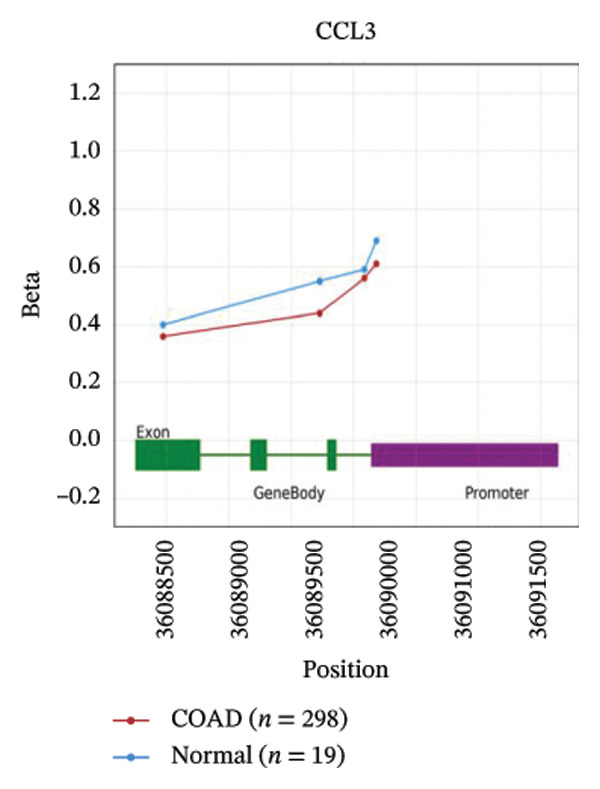
(a)

**Figure:**
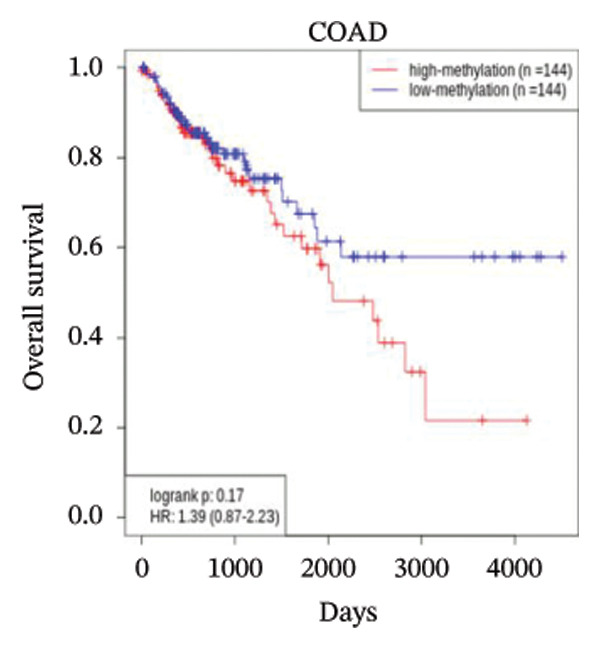
(b)

**Figure:**
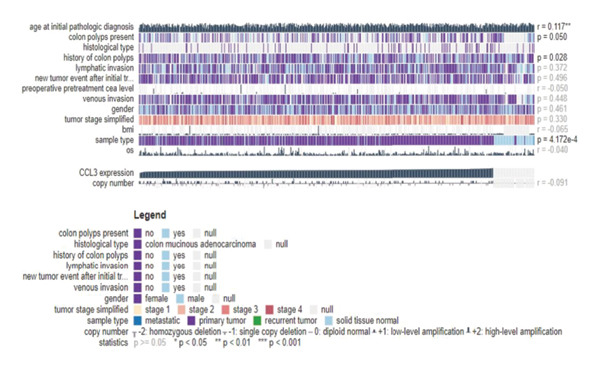
(c)

**Figure:**
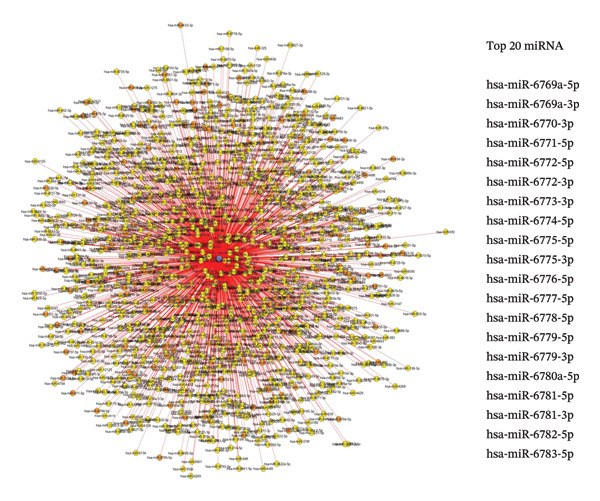
(d)

### 3.9. Identification of miRNAs Regulating CCL3

miRWalk was used to screen for miRNAs targeting CCL3 and to construct the miRNA–gene network. Figure 8(d) shows CCL3 as a target and a network of 1000 miRNAs. The number of lines indicates the correlation between miRNAs. The top 20 miRNAs that may target CCL3 are shown in Figure 8(d).

## 4. Discussion

Several studies have reported high CCL3 expression in different cancers [15–21]. CCL3 is highly expressed in colorectal cancer, promotes cell proliferation, and is closely related to the TRAF6/NF‐κB pathway [35]. This study demonstrates that high CCL3 expression is an independent adverse prognostic factor in COAD, correlates with immune infiltration, and potentially influences treatment response. However, a previous study using cytokine microarray analysis showed that CCL3 mediates the interaction between intrahepatic cholangiocarcinoma and hepatocytes, thereby enhancing hepatocyte migration and invasion [36]. A study reported that high CCL3 expression in tumor antigen‐presenting cells and cancer cells in COAD activates the Akt signaling pathway and facilitates cell migration and invasiveness, contributing to poor prognosis in patients with COAD [25]. Furthermore, we examined three datasets, GEPIA2, UALCAN, and TIMER, which compile gene expression data from thousands of tumors and normal tissue samples. The results indicated that CCL3 expression was higher in various cancers than in normal tissues. Moreover, the higher expression of CCL3 in later stages and its association with nodal metastasis and TP53 mutations suggest a potential role for CCL3 in promoting tumor progression and metastasis in OSCC. Increased CCL3 expression was associated with poor OS and RFS in colon cancer, SCC, and thymoma. Our survival analysis confirmed that CCL3 remained significant in the univariate analysis (HR = 1.52) and independent after multivariate adjustment (aHR = 1.42), highlighting its role as a robust prognostic indicator. However, immune cell infiltration is a critical factor in the progression, prognosis, and treatment response of various cancers. TCGA analyses (Tables 2 and 3) confirm CCL3’s strong correlation between M2‐polarized TAMs (Cor = 0.72) and neutrophils, fostering an immunosuppressive tumor immune microenvironment (TIME) via markers such as IL10 and HAVCR2. However, Wang et al. demonstrated that in microsatellite‐stable (MSS) colorectal cancer, PIN1 promotes tumor growth and immune evasion by activating NF‐κB signaling and the CCL3–CCR5 axis, thereby enhancing regulatory T‐cell recruitment and cancer‐associated fibroblast activation. The same study also suggested that inhibiting PIN1 reduces CCL3‐mediated immunosuppression and improves the efficacy of anti‐PD‐1 therapy [37]. Similarly, Su et al. identified CCR1^+^ macrophages as key regulators of immune checkpoint blockade (ICB) response in melanoma, with the CCR1–CCL3 axis driving the formation of immunosuppressive niches that support CD8^+^ exhausted T cells; pharmacological CCR1 blockade enhanced anti‐PD‐1 efficacy [38]. Pietrobono et al. reported that in pancreatic ductal adenocarcinoma (PDAC), baseline plasma CCL3 levels predict responses to TGFβ receptor inhibition with galunisertib. High CCL3 levels indicated poor prognosis in placebo‐treated patients but improved survival when combined with gemcitabine. Mechanistically, tumor‐derived CCL3 activates TGFβ signaling in macrophages, promoting M2 polarization and Lif secretion, sustaining a mesenchymal/basal‐like tumor phenotype. Galunisertib redirects macrophages to the M1 phenotype, reduces Lif, and shifts PDAC cells toward an epithelial/classical state, enhancing gemcitabine sensitivity [39]. Moreover, in COAD, Guan et al. demonstrated that CCL3 is highly expressed in both TAMs and cancer cells. CCL3 binds to CCR5 and activates the Akt pathway to enhance tumor cell migration and invasiveness. TAM‐ and tumor‐derived CCL3 collaboratively drive malignancy, and high CCL3/CCR5 expression correlates with a poor prognosis [25]. Additionally, Li et al. reported that several CC chemokines, including CCL3, are downregulated in urothelial BC, with chemokine expression correlating with the pathological stage and immune cell infiltration. High levels of multiple CC chemokines are associated with improved prognosis, highlighting their potential role in BC progression and tumor immunity [40]. In Waldenström macroglobulinemia (WM), a study found that elevated serum CCL3 and sRANKL levels predicted worse outcomes. High sRANKL levels shortened OS, whereas elevated CCL3 levels predicted shorter progression‐free survival. WM cells strongly express CCL3, which may stimulate RANKL production in the bone microenvironment, supporting the rationale for targeting CCL3 and RANKL in preclinical models [41]. Furthermore, a study done by Takahashi et al. demonstrated in DLBCL that BCR signaling triggers CCL3 and CCL4 secretion, recruiting accessory cells. High serum levels of these chemokines correlate with adverse clinical features and shorter survival, particularly in activated B cell–like DLBCL, and are sensitive to BTK inhibition, establishing CCL3/CCL4 as biomarkers for BCR activation and prognosis [22]. In CLL, Sivina et al. showed that BCR‐driven CCL3 secretion promotes interactions with the leukemia microenvironment. In 351 patients, plasma CCL3 levels correlated with prognostic markers, including ZAP‐70, CD38, IGHV mutation, and cytogenetics. High CCL3 levels were independently associated with a shorter time to treatment, highlighting its utility as a prognostic biomarker [42]. Collectively, these studies underscore that CCL3, often acting via the CCL3–CCR5 axis, plays a central role in tumor progression, immune modulation, and therapy responsiveness across multiple malignancies, making it a promising prognostic biomarker and potential therapeutic target.

Our study found a strong positive correlation between CCL3 expression and immune infiltration in COAD. CCL3 expression was significantly associated with the infiltration of CD + T cells, macrophages, neutrophils, and DCs. Immune cell infiltration in COAD plays a crucial role in shaping the TME, influencing tumor progression, and affecting patient prognosis. The immune landscape of COAD is also key in determining the efficacy of immunotherapies, making immune profiling an important tool for personalized cancer treatment [43–46]. However, Wang et al. demonstrated that in renal carcinoma, high mitochondrial antiviral signaling protein (MAVS) expression correlates with poor prognosis and reduced CD8^+^ T‐cell infiltration. Loss of MAVS disrupts mitochondrial homeostasis via CMTM6 destabilization, resulting in mitochondrial dysfunction, reactive oxygen species accumulation, and cellular senescence. This senescent state induces a chemokine‐rich secretory phenotype dominated by CCL3, which recruits and activates CD8^+^ T cells, suppressing tumor growth and enhancing responsiveness to PD‐1 blockade [47]. These findings highlight the MAVS–CMTM6–CCL3 axis as a promising therapeutic target for renal carcinoma. Yuan et al. identified CCL3 as a central driver of intestinal injury in necrotizing enterocolitis (NEC). Mechanistically, CCL3 signals through CCR4, activating the ERK1/2–NF‐κB pathway to regulate apoptosis via BAX/BCL‐2 expression. Inhibition of CCR4, ERK1/2, or NF‐κB reversed CCL3‐mediated injury, positioning CCL3 as a key pathogenic mediator and therapeutic target in NEC [48]. In another study, Baba et al. demonstrated that CCL3 mediates basophil‐driven leukemogenesis in chronic myeloid leukemia (CML). Basophils constitutively produce CCL3 in the bone marrow, suppressing normal hematopoiesis, particularly during stem cell reconstitution after transplantation [49]. In CML, CCL3‐expressing basophil‐like leukemia cells accumulate in the bone marrow, creating a leukemia‐permissive niche that selectively supports leukemia‐initiating cell expansion while inhibiting normal progenitor cells. This mechanism identifies basophil‐derived CCL3 as a therapeutic target for CML [49]. Moreover, Ramos et al. established CCL3 as the principal chemokine mediating neutrophil recruitment during murine immune inflammation. Neutrophil migration was markedly impaired by ovalbumin‐induced CCL3 depletion but remained unaffected by CCL4 or CCL5 blockade [50]. Mechanistically, CCL3 signals through CCR1, initiating a sequential inflammatory cascade involving TNF‐α and leukotriene B4 (LTB4), both of which are required for effective neutrophil recruitment [50]. These findings position CCL3–CCR1 signaling as a key regulator of immune‐mediated neutrophil trafficking and a potential therapeutic target for inflammatory conditions.

The study also analyzed tumor infiltration levels in COAD with different SCNAs of CCL3 and found that arm‐level gain in B cells, deep deletion and arm‐level gain in CD8+ T cells, deep deletion and arm‐level gain in neutrophils, and arm‐level gain in DCs significantly contributed to infiltration. CCL3 promotes immune cell infiltration in the TME through a complex mechanism involving various immune cell types and signaling pathways. CCL3, expressed on neutrophils, guides immune cell infiltration by binding to chemokine receptors (CCR1 and CCR5) expressed on immune cells [51].

Our exploratory analysis using TCGA‐COAD subsets (*n* = 128) showed that high CCL3 expression was associated with lower complete response rates (18% vs. 32%, *p* 0.041) and higher rates of progressive disease (25% vs. 14%). CCL3 has been frequently highlighted in multiple cancer studies and database analyses [39, 46, 52]. However, Zhou et al. used single‐cell RNA sequencing to compare lesion tissues with normal breast tissues and characterize the immune landscape in granulomatous mastitis (GM). GM tissues exhibited pronounced immune cell infiltration and immune imbalance, including increased Th1 cells, enrichment of Toll‐like receptor signaling, and elevated expression of inflammatory mediators such as IFN‐γ, CCL3, CCL4, CXCL13, CD69, STAT1, and HSPA1A. Macrophage populations displayed a shift toward a proinflammatory phenotype with the activation of IFN‐γ, IFN‐α, IL‐6/JAK/STAT3, and TNF‐α/NF‐κB pathways [53]. A study done by Li et al. identified a novel circular RNA, circPRKAR1B, that is upregulated in esophageal squamous cell carcinoma (ESCC) and is associated with poor patient prognosis. Functional analyses showed that circPRKAR1B interacts with pyruvate kinase M2 (PKM2), leading to the activation of the NF‐κB signaling pathway and increased secretion of CCL3, thereby promoting tumor progression [54].

We also investigated the coexpression network of CCL3 in COAD to identify associated biological processes and pathways. The coexpressed genes were primarily involved in biological processes such as immune tolerance induction, amyloid beta clearance, interleukin 4 production, NADH dehydrogenase complex assembly, mitochondrial respiratory chain complex assembly, and peroxisome organization. Notably, interleukin‐4 production is associated with the polarization of macrophages to the M2 phenotype, which promotes tumor progression and metastasis [55, 56]. Furthermore, KEGG pathway analysis showed that most CCL3‐correlated genes were associated with aldarate metabolism, glycosylphosphatidylinositol‐anchor biosynthesis, and pyruvate metabolism. GPI‐anchored proteins are essential for cell‐to‐cell interactions and signal transduction across the cell membrane. GPI‐anchored proteins may affect the ability of T cells to regulate their immunological responses through their interactions with immune regulatory factors. Disruption of these interactions by abnormal GPI‐anchored protein biosynthesis may impact T‐cell activation, proliferation, and effector function [57]. These findings further strengthen the potential role of CCL3 in TME regulation, tumor progression, and metastasis in COAD.

CCL3 is emerging as a critical regulator of the TIME and disease progression across diverse malignancies. Liu et al. demonstrated that in hepatocellular carcinoma (HCC), CCL3 is downregulated and positively correlates with immune infiltration and inflammatory signatures. Hepatic delivery of rAAV‐Ccl3 reprograms the TIME, enhancing macrophage antigen uptake, MHC‐II expression, CD8^+^ T‐cell infiltration, and formation of tertiary lymphoid structures, ultimately strengthening adaptive immunity and improving responsiveness to ICB [58]. Transcriptional regulation of CCL3 also contributes to immune remodeling. Song et al. showed that RUNX3 in lung adenocarcinoma promotes CD8^+^ T‐cell recruitment through the upregulation of CCL3 and CCL20 [59], while Liang et al. reported that hypoxia in intrahepatic cholangiocarcinoma drives M2 polarization of APOE^+^ TAMs and recruitment of regulatory T cells via the CCL3–CCR5 axis, establishing an immunosuppressive niche [60]. In pancreatic cancer, Zhang et al. revealed that Treg–fibroblast crosstalk regulates CCL3 expression, driving myeloid recruitment and restoring immune suppression after Treg depletion [61]. Similarly, Pitarresi et al. found that fibroblast‐expressed ETS2 regulates CCL3 and CCL4, shaping immunosuppressive microenvironments during acinar‐to‐ductal metaplasia in early pancreatic tumorigenesis [62].

Moreover, in renal carcinoma, Yeh et al. reported that infiltrating T cells induce CCL3 secretion via ERβ signaling, promoting tumor invasiveness and creating a positive feedback loop that further recruits T cells. MoDC‐derived CCL chemokines also regulate immune cell recruitment [63]. Kang et al. showed that CCL17 and CCL22 mediate Treg attraction, and their silencing enhances CD8^+^ T‐cell infiltration and antitumor immunity [64]. In glioblastoma, RNF135^+^ TAMs utilize the CCL3–CCR1 axis to mediate TAM–tumor crosstalk, increasing T‐cell infiltration and predicting sensitivity to MEK inhibition [65]. Mechanistically, tumor–macrophage metabolic crosstalk modulates CCL3. In TNBC, CCL3^+^ macrophages are depleted via glutamine competition with HEBP2‐high tumor cells, weakening antitumor immunity, whereas GSTP1 inhibition restores CCL3^+^ macrophage function and sensitizes tumors to immunotherapy [66]. In OV, the β‐catenin–metadherin/CEACAM1–CCL3 axis, where TAMs induce CCL3 to promote polyploidization and metastasis, highlights a positive feedback loop driving tumor aggressiveness [67]. Additional studies reinforce CCL3’s central role of in malignancies. A study reported that tumor‐induced CCL3 promotes MDSC differentiation via CCR1/CCR5 signaling and silencing these receptors repolarizes MDSCs into tumoricidal neutrophils [68]. In the context of colorectal cancer, a study found that CCL3 and CCL4 levels are elevated in colorectal cancer, correlating with pro‐tumor TAM infiltration and desmoplastic progression [18]. Overall, these findings illustrate that CCL3 regulates immune cell recruitment, tumor–stromal interactions, and microenvironmental remodeling, acting as both a prognostic biomarker and therapeutic target across cancers.

Methylation analysis has shown that CCL3 often undergoes hypermethylation in gene body regions that are close to the promoter. Notably, this hypermethylation of CCL3 is linked to reduced survival rates in patients with COAD, indicating that epigenetic silencing of the gene may play a role in tumor progression. These findings are preliminary and provide a basis for future research on epigenetic therapies that regulate CCL3 activity and enhance clinical outcomes in COAD. For instance, a recent study explored the impact of dual inhibitors targeting DNA methyltransferases (DNMTs) and histone deacetylases (HDACs) on colorectal cancer. The results indicate that these inhibitors can alter the immune microenvironment of tumors, thereby improving the effectiveness of anti‐PD‐L1 therapy. This study underscores the potential of combining epigenetic modulation with immune checkpoint inhibition to enhance colorectal cancer treatment outcomes [69]. In its role in therapeutic response, recent studies have highlighted CCL3 as a critical mediator with prognostic value across diverse malignancies. A study identified plasma CCL3, along with macrophage migration inhibitory factor (MIF), as a novel biomarker for nasopharyngeal carcinoma (NPC). Elevated CCL3 levels distinguished NPC patients from EBV‐positive and EBV‐negative controls, with a combined biomarker panel achieving high diagnostic accuracy (AUC 0.961) [70], suggesting CCL3 as a sensitive indicator of early disease in high‐risk populations. In hematologic malignancies, Shcherbina et al. demonstrated that malignant B cells in CLL with CD150/CD180 expression exhibit high CCL3 and CCL4 levels, which correlates with increased sensitivity to bendamustine. Basal CCL3/CCL4 levels serve as predictors of chemotherapy response, linking chemokine profiles to personalized therapeutic strategies [71]. CCL3 can also be used to predict immunotherapy outcomes. Zhang et al. developed a liquid biopsy–based model in HNSCC, where nonresponders to neoadjuvant PD‐1 therapy exhibited elevated plasma CCL3 and CCL4. Integrating peripheral blood immune subsets and cytokine profiles achieved high predictive accuracy (AUC = 0.9219), highlighting CCL3’s utility as a blood‐based biomarker for therapy stratification [72]. Similarly, Yang et al. reported that TACC2 overexpression in soft tissue sarcoma enhances CCL3 transcription, promoting CD8^+^ T‐cell infiltration and synergizing with anti‐PD‐1 therapy, suggesting that CCL3 is a mechanistic link between transcriptional regulation and immunotherapy responsiveness [73]. In addition, CCL3 functions as a chemotactic and immunomodulatory factor. Mitchell et al. demonstrated that DC vaccine efficacy in glioblastoma is enhanced by tetanus/diphtheria recall‐antigen preconditioning via CCL3‐mediated DC migration and tumor suppression [74]. Another study showed that CCL3 activates CCR5‐dependent signaling in CD4^+^ T cells, upregulating CD40L, maturing antigen‐presenting cells, and enhancing CD8^+^ T‐cell priming and tumor infiltration. CCR5‐mediated CCL3 signaling reduces chemically induced fibrosarcoma growth and is essential for TLR9‐mediated antitumor reactivation, underscoring the role of this chemokine in coordinating effective antitumor immunity [75]. Overall, these studies illustrate that CCL3 is a multifaceted mediator in cancer, acting as a biomarker for diagnosis, therapy response, and prognosis, while functionally modulating immune cell recruitment, DC migration, and CD8^+^ T‐cell activation. Notably, high CCL3 levels may also reflect immunosuppressive microenvironments, identifying patients at risk of therapy resistance and providing a rationale for combination strategies targeting the CCL3–CCR axis in addition to chemotherapy, targeted therapy, or ICB.

This study has several limitations that should be considered in future research aimed at identifying genes or markers. One limitation of this study is that our findings rely solely on in silico analysis, without validation through laboratory tests such as immunohistochemistry, PCR, or experiments involving cells or animals. Consequently, we could not definitively establish cause‐and‐effect relationships. Additionally, we utilized public datasets (TCGA, GTEx), which may contain biases stemming from variations in sample collection, sequencing methods, and patient characteristics. Despite employing rigorous statistical methods, challenges such as multiple testing, batch effects, and dataset discrepancies may persist. Nevertheless, our study provides an in‐depth examination of the association between CCL3 expression, immune infiltration, and patient outcomes. This study indicates that CCL3 can function as both a biomarker and a target for modulating the immune system. To validate these findings and explore CCL3’s potential as a therapeutic target or biomarker in immunotherapy, further laboratory and clinical investigations are essential. Additionally, functional studies in COAD cell lines, including miRNAs that directly regulate CCL3 in COAD and the knockdown or overexpression of CCL3, will be vital to determine the causal effects and clarify its role in tumor immunity. These efforts could help translate our in silico approaches into actionable insights and potential therapeutic strategies.

## 5. Conclusion

This study showed that CCL3 is associated with cancer progression, particularly in COAD. CCL3 is an independent prognostic biomarker in COAD, shaping an immunosuppressive TIME and linking therapy resistance. Its expression in various cancers, specifically in COAD, is correlated with advanced stages, lymph node involvement, and TP53 mutations. Our analysis revealed that CCL3 levels serve as prognostic indicators: Elevated levels were associated with poor survival in colon cancer, SCC, and thymoma, whereas reduced levels were associated with poor survival in BLCA, KIRP, UCEC, and KIRC. Furthermore, CCL3 levels correlated with the presence of immune cells, including CD8+ T cells, M2 macrophages, and MDSCs. Targeting CCL3 could enhance the efficacy of immunotherapy and chemotherapy; however, further research and validation in prospective cohorts are needed to elucidate the underlying mechanisms.

## Author Contributions

All authors made significant contributions to the work reported, whether in the conception, study design, execution, acquisition of data, analysis, and interpretation, or in all these areas; and participated in drafting, revising, or critically reviewing the article.

## Funding

This study received no external funding.

## Disclosure

All authors gave the final approval of the version to be published; agreed on the journal to which the article was submitted; and agreed to be accountable for all aspects of the work.

## Conflicts of Interest

The authors declare no conflicts of interest.

## Data Availability

The original contributions presented in this study are included in this article. Further inquiries should be directed to the corresponding author.

## References

[1] Bray F. , Laversanne M. , Sung H. et al., Global Cancer Statistics 2022: GLOBOCAN Estimates of Incidence and Mortality Worldwide for 36 Cancers in 185 Countries, CA: A Cancer Journal for Clinicians. (2024) 74, no. 3, 229–263, 10.3322/caac.21834. 38572751

[2] Worthley D. L. and Leggett B. A. , Colorectal Cancer: Molecular Features and Clinical Opportunities, Clinical Biochemist Reviews. (2010) 31, no. 2, 31–38. 20498827 PMC2874430

[3] Dawson H. , Kirsch R. , Messenger D. , and Driman D. , A Review of Current Challenges in Colorectal Cancer Reporting, Archives of Pathology and Laboratory Medicine. (2019) 143, no. 7, 869–882, 10.5858/arpa.2017-0475-RA, 2-s2.0-85068564467. 30672337

[4] Wells K. O. and Senagore A. , Minimally Invasive Colon Cancer Surgery, Surgical Oncology Clinics of North America. (2019) 28, no. 2, 285–296, 10.1016/j.soc.2018.11.004, 2-s2.0-85058993866. 30851829

[5] Liu Q. , Huang Y. , Luo D. et al., Evaluating the Guiding Role of Elevated Pretreatment Serum Carcinoembryonic Antigen Levels for Adjuvant Chemotherapy in Stage IIA Colon Cancer: A Large Population-Based and Propensity Score-Matched Study, Frontiers in Oncology. (2019) 9, 10.3389/fonc.2019.00037, 2-s2.0-85063322971. PMC6381003 30815388

[6] Ven Fong Z. , Chang D. C. , Lillemoe K. D. , Nipp R. D. , Tanabe K. K. , and Qadan M. , Contemporary Opportunity for Prehabilitation as Part of an Enhanced Recovery After Surgery Pathway in Colorectal Surgery, Clinics in Colon and Rectal Surgery. (2019) 32, 95–101, 10.1055/s-0038-1676473, 2-s2.0-85062340755. 30833857 PMC6395100

[7] Schaad N. , Berezowska S. , Perren A. , and Hewer E. , Impact of Template-Based Synoptic Reporting on Completeness of Surgical Pathology Reports, Virchows Archiv: An International Journal of Pathology. (2024) 484, no. 1, 31–36, 10.1007/s00428-023-03533-6. 37017774 PMC10791929

[8] Neophytou C. M. , Panagi M. , Stylianopoulos T. , and Papageorgis P. , The Role of Tumor Microenvironment in Cancer Metastasis: Molecular Mechanisms and Therapeutic Opportunities, Cancers. (2021) 13, no. 9, 10.3390/cancers13092053. PMC8122975 33922795

[9] Hussain S. , Peng B. , Cherian M. , Song J. W. , Ahirwar D. K. , and Ganju R. K. , The Roles of Stroma-Derived Chemokine in Different Stages of Cancer Metastases, Frontiers in Immunology. (2020) 11, 10.3389/fimmu.2020.598532. PMC7783453 33414786

[10] Nagarsheth N. , Wicha M. S. , and Zou W. , Chemokines in the Cancer Microenvironment and Their Relevance in Cancer Immunotherapy, Nature Reviews Immunology. (2017) 17, no. 9, 559–572, 10.1038/nri.2017.49, 2-s2.0-85028431759. PMC5731833 28555670

[11] Heo S. C. , Nam I. H. , Keum B. R. , Yun Y. G. , Lee J. Y. , and Kim H. J. , C-X-C Motif Chemokine Ligand 1 Derived From Oral Squamous Cell Carcinoma Promotes Cancer-Associated Fibroblast Differentiation and Tumor Growth, Molecular biomedicine. (2025) 6, no. 1, 10.1186/s43556-025-00281-8. PMC12149066 40490643

[12] Raman D. , Baugher P. J. , Thu Y. M. , and Richmond A. , Role of Chemokines in Tumor Growth, Cancer Letters. (2007) 256, no. 2, 137–165, 10.1016/j.canlet.2007.05.013, 2-s2.0-34548665711. 17629396 PMC2065851

[13] Prakash J. and Shaked Y. , The Interplay Between Extracellular Matrix Remodeling and Cancer Therapeutics, Cancer Discovery. (2024) 14, no. 8, 1375–1388, 10.1158/2159-8290.CD-24-0002. 39091205 PMC11294818

[14] Korbecki J. , Grochans S. , Gutowska I. , Barczak K. , and Baranowska-Bosiacka I. , CC Chemokines in a Tumor: A Review of Pro-Cancer and Anti-Cancer Properties of Receptors CCR5, CCR6, CCR7, CCR8, CCR9, and CCR10 Ligands, International Journal of Molecular Sciences. (2020) 21, no. 20, 10.3390/ijms21207619. PMC7590012 33076281

[15] Schaller T. H. , Batich K. A. , Suryadevara C. M. , Desai R. , and Sampson J. H. , Chemokines as Adjuvants for Immunotherapy: Implications for Immune Activation With CCL3, Expert Review of Clinical Immunology. (2017) 13, no. 11, 1049–1060, 10.1080/1744666X.2017.1384313, 2-s2.0-85031424726. 28965431 PMC6020048

[16] Rajagopal S. , Bassoni D. L. , Campbell J. J. , Gerard N. P. , Gerard C. , and Wehrman T. S. , Biased Agonism as a Mechanism for Differential Signaling by Chemokine Receptors, Journal of Biological Chemistry. (2013) 288, no. 49, 35039–35048, 10.1074/jbc.M113.479113, 2-s2.0-84890282799. 24145037 PMC3853256

[17] Balkwill F. , Cancer and the Chemokine Network, Nature Reviews Cancer. (2004) 4, no. 7, 540–550, 10.1038/nrc1388, 2-s2.0-3042822267. 15229479

[18] De la Fuente López M. , Landskron G. , Parada D. et al., The Relationship Between Chemokines CCL2, CCL3, and CCL4 With the Tumor Microenvironment and Tumor-Associated Macrophage Markers in Colorectal Cancer, Tumour Biology: The Journal of the International Society for Oncodevelopmental Biology and Medicine. (2018) 40, 10.1177/1010428318810059, 2-s2.0-85056325625. 30419802

[19] Chanmee T. , Ontong P. , Konno K. , and Itano N. , Tumor-Associated Macrophages as Major Players in the Tumor Microenvironment, Cancers. (2014) 6, no. 3, 1670–1690, 10.3390/cancers6031670, 2-s2.0-84920943285. 25125485 PMC4190561

[20] Bone Marrow-Derived Mesenchymal Stem Cells Promote Colorectal Cancer Progression Through Paracrine Neuregulin 1/HER3 Signalling-PubMed, https://pubmed.ncbi.nlm.nih.gov/22535374/. 10.1136/gutjnl-2011-301393 22535374

[21] CCL3 (MIP-1α) Plasma Levels and the Risk for Disease Progression in Chronic Lymphocytic Leukemia-PubMed, https://pubmed.ncbi.nlm.nih.gov/21115978/. 10.1182/blood-2010-09-307249 PMC3318778 21115978

[22] Takahashi K. , Sivina M. , Hoellenriegel J. et al., CCL3 and CCL4 are Biomarkers for B Cell Receptor Pathway Activation and Prognostic Serum Markers in Diffuse Large B Cell Lymphoma, British Journal of Haematology. (2015) 171, no. 5, 726–735, 10.1111/bjh.13659, 2-s2.0-84983133251. 26358140 PMC4715651

[23] Circulating Soluble Receptor Activator of Nuclear Factor Kappa B Ligand and C-C Motif Ligand 3 Correlate With Survival in Patients With Waldenström Macroglobulinemia-PubMed, https://pubmed.ncbi.nlm.nih.gov/29685422/. 10.1016/j.clml.2018.03.010 29685422

[24] Liu L. , Yu Z. , Cheng H. et al., Multiple Myeloma Hinders Erythropoiesis and Causes Anaemia Owing to High Levels of CCL3 in the Bone Marrow Microenvironment, Scientific Reports. (2020) 10, no. 1, 10.1038/s41598-020-77450-y. PMC7689499 33239656

[25] Guan B. , Li H. , Yao J. et al., CCL3-CCR5 Axis Promotes Cell Migration and Invasion of Colon Adenocarcinoma Via Akt Signaling Pathway, Environmental Toxicology. (2023) 38, no. 1, 172–184, 10.1002/tox.23675. 36346222

[26] Tang Z. , Kang B. , Li C. , Chen T. , and Zhang Z. , GEPIA2: An Enhanced Web Server for Large-Scale Expression Profiling and Interactive Analysis, Nucleic Acids Research. (2019) 47, no. 1, W556–W560, 10.1093/nar/gkz430, 2-s2.0-85069235274. 31114875 PMC6602440

[27] Chandrashekar D. S. , Karthikeyan S. K. , Korla P. K. et al., UALCAN: An Update to the Integrated Cancer Data Analysis Platform, Neoplasia. (2022) 25, 18–27, 10.1016/j.neo.2022.01.001. 35078134 PMC8788199

[28] Li T. , Fan J. , Wang B. et al., TIMER: A Web Server for Comprehensive Analysis of Tumor-Infiltrating Immune Cells, Cancer Research. (2017) 77, no. 21, e108–e110, 10.1158/0008-5472.CAN-17-0307, 2-s2.0-85035064069. 29092952 PMC6042652

[29] Győrffy B. , Transcriptome-Level Discovery of Survival-Associated Biomarkers and Therapy Targets in Non-Small-Cell Lung Cancer, British Journal of Pharmacology. (2024) 181, no. 3, 362–374, 10.1111/bph.16257. 37783508

[30] Vasaikar S. V. , Straub P. , Wang J. , and Zhang B. , LinkedOmics: Analyzing Multi-Omics Data Within and Across 32 Cancer Types, Nucleic Acids Research. (2018) 46, no. D1, D956–D963, 10.1093/nar/gkx1090, 2-s2.0-85040936922. 29136207 PMC5753188

[31] Ru B. , Wong C. N. , Tong Y. et al., TISIDB: An Integrated Repository Portal for TUMOR-Immune System Interactions, Bioinformatics (Oxford. Online). (2019) 35, no. 20, 4200–4202, 10.1093/bioinformatics/btz210, 2-s2.0-85071048049. 30903160

[32] Tang G. , Cho M. , and Wang X. , OncoDB: An Interactive Online Database for Analysis of Gene Expression and Viral Infection in Cancer, Nucleic Acids Research. (2022) 50, no. 1, D1334–D1339, 10.1093/nar/gkab970. 34718715 PMC8728272

[33] Koch A. , De Meyer T. , Jeschke J. , and Van Criekinge W. , MEXPRESS: Visualizing Expression, DNA Methylation and Clinical TCGA Data, BMC Genomics. (2015) 16, no. 1, 10.1186/s12864-015-1847-z, 2-s2.0-84940204580. PMC4549898 26306699

[34] Sticht C. , De La Torre C. , Parveen A. , and Gretz N. , miRWalk: An Online Resource for Prediction of microRNA Binding Sites, PLoS One. (2018) 13, no. 10, 10.1371/journal.pone.0206239, 2-s2.0-85055080177. PMC6193719 30335862

[35] Ma X. , Su J. , Zhao S. et al., CCL3 Promotes Proliferation of Colorectal Cancer Related With TRAF6/NF-κB Molecular Pathway, Contrast Media and Molecular Imaging. (2022) 2022, no. 1, 10.1155/2022/2387192. PMC9296340 35935327

[36] Zhou S. , Yang K. , Chen S. et al., CCL3 Secreted by Hepatocytes Promotes the Metastasis of Intrahepatic Cholangiocarcinoma by VIRMA-Mediated N6-Methyladenosine (m6A) Modification, Journal of Translational Medicine. (2023) 21, no. 1, 10.1186/s12967-023-03897-y. PMC9869516 36691046

[37] Wang J. , Tang S. , Fan J. et al., Targeting Pin1 to Overcome Immunosuppressive Tumor Microenvironment in MSS Colorectal Cancer, Frontiers in Immunology. (2025) 16, 10.3389/fimmu.2025.1677029. PMC12615396 41246306

[38] Su X. , Huang R. , Kang D. , Wang J. , Li L. , and Zou Z. , Blocking CCR1^+^ Macrophages Overcomes Resistance to Immune Checkpoint Inhibitors in Melanoma, Cell Communication and Signaling: CCS. (2025) 23, no. 1, 10.1186/s12964-025-02444-0. PMC12581358 41184864

[39] Pietrobono S. , Bertolini M. , De Vita V. et al., CCL3 Predicts Exceptional Response to TGFβ Inhibition in Basal-Like Pancreatic Cancer Enriched in LIF-Producing Macrophages, npj Precision Oncology. (2024) 8, no. 1, 10.1038/s41698-024-00742-3. PMC11525688 39478186

[40] Li Y. , Chen X. , Li D. et al., Identification of Prognostic and Therapeutic Value of CC Chemokines in Urothelial Bladder Cancer: Evidence From Comprehensive Bioinformatic Analysis, BMC Urology. (2021) 21, no. 1, 10.1186/s12894-021-00938-w. PMC8665633 34893045

[41] Eleutherakis-Papaiakovou E. , Kastritis E. , Gavriatopoulou M. et al., Circulating Soluble Receptor Activator of Nuclear Factor Kappa B Ligand and C-C Motif Ligand 3 Correlate With Survival in Patients With Waldenström Macroglobulinemia, Clinical Lymphoma, Myeloma & Leukemia. (2018) 18, no. 6, 431–437, 10.1016/j.clml.2018.03.010, 2-s2.0-85045832914. 29685422

[42] Sivina M. , Hartmann E. , Kipps T. J. et al., CCL3 (MIP-1α) Plasma Levels and the Risk for Disease Progression in Chronic Lymphocytic Leukemia, Blood. (2011) 117, no. 5, 1662–1669, 10.1182/blood-2010-09-307249, 2-s2.0-79551629508. 21115978 PMC3318778

[43] Galon J. , Costes A. , Sanchez-Cabo F. et al., Type, Density, and Location of Immune Cells Within Human Colorectal Tumors Predict Clinical Outcome, Science. (2006) 313, no. 5795, 1960–1964, 10.1126/science.1129139, 2-s2.0-33749319703. 17008531

[44] Tosolini M. , Kirilovsky A. , Mlecnik B. et al., Clinical Impact of Different Classes of Infiltrating T Cytotoxic and Helper Cells (Th1, th2, treg, th17) in Patients With Colorectal Cancer, Cancer Research. (2011) 71, no. 4, 1263–1271, 10.1158/0008-5472.CAN-10-2907, 2-s2.0-79951815749. 21303976

[45] Overman M. J. , McDermott R. , Leach J. L. et al., Nivolumab in Patients With Metastatic DNA Mismatch Repair-Deficient or Microsatellite Instability-High Colorectal Cancer (CheckMate 142): An Open-Label, Multicentre, Phase 2 Study, The Lancet Oncology. (2017) 18, no. 9, 1182–1191, 10.1016/S1470-2045(17)30422-9, 2-s2.0-85025439295. 28734759 PMC6207072

[46] Mlecnik B. , Bindea G. , Angell H. K. et al., Integrative Analyses of Colorectal Cancer Show Immunoscore is a Stronger Predictor of Patient Survival Than Microsatellite Instability, Immunity. (2016) 44, no. 3, 698–711, 10.1016/j.immuni.2016.02.025, 2-s2.0-84960466507. 26982367

[47] Wang H. , Fan Y. , Liang Q. et al., MAVS/CMTM6 Axis Couples Mitochondrial Homeostasis to Immunogenic Senescence Via CCL3-Driven T-Cell Recruitment in Renal Carcinoma, Journal for Immunotherapy of Cancer. (2025) 13, no. 12, 10.1136/jitc-2025-011477. PMC12766799 41469142

[48] Yuan X. , Xiong Z. , Liu W. et al., Novel Therapeutic Targeting of CCL3-CCR4 Axis Mediated Apoptotic Intesitnal Injury in Necrotizing Enterocolitis, Frontiers in Immunology. (2022) 13, 10.3389/fimmu.2022.859398. PMC9073010 35529858

[49] Baba T. , Tanabe Y. , Yoshikawa S. et al., MIP-1α/CCL3-Expressing Basophil-Lineage Cells Drive the Leukemic Hematopoiesis of Chronic Myeloid Leukemia in Mice, Blood. (2016) 127, no. 21, 2607–2617, 10.1182/blood-2015-10-673087, 2-s2.0-84982792381. 27006388

[50] Ramos C. D. , Canetti C. , Souto J. T. et al., MIP-1alpha[CCL3] Acting on the CCR1 Receptor Mediates Neutrophil Migration in Immune Inflammation Via Sequential Release of TNF-Alpha and LTB4, Journal of Leukocyte Biology. (2005) 78, no. 1, 167–177, 10.1189/jlb.0404237, 2-s2.0-22144465474. 15831559

[51] Proudfoot A. E. I. , Chemokine Receptors: Multifaceted Therapeutic Targets, Nature Reviews Immunology. (2002) 2, 106–115, 10.1038/nri722, 2-s2.0-0036485214. PMC7097668 11910892

[52] Guo X. , Sun Z. , Liang Y. et al., Unraveling the Crucial Role of CCL3 in Nasopharyngeal Carcinoma: Bioinformatics and Immunohistochemical Insights, Journal of Pathology and Translational Medicine. (2025) 59, no. 5, 281–290, 10.4132/jptm.2025.05.23. 40916884 PMC12455464

[53] Zhou Y. , Deng X. , Ruan H. et al., Single-Cell RNA Sequencing Reveals the Immune Landscape of Granulomatous Mastitis, Inflammation. (2025) 48, no. 6, 4046–4061, 10.1007/s10753-025-02310-8. 40338490 PMC12722302

[54] Li S. , Ma J. , Jia W. et al., EIF4A3-Induced circPRKAR1B Promotes Esophageal Squamous Cell Carcinoma Progression Through Binding PKM2 to Regulate NF-κB Induced CCL3 Secretion, Cancer Cell International. (2025) 25, no. 1, 10.1186/s12935-025-03905-9. PMC12269245 40671099

[55] Martinez F. O. , Sica A. , Mantovani A. , and Locati M. , Macrophage Activation and Polarization, Frontiers in Bioscience: A Journal and Virtual Library. (2008) 13, 453–461, 10.2741/2692, 2-s2.0-38449102676. 17981560

[56] DeNardo D. G. , Barreto J. B. , Andreu P. et al., CD4(+) T Cells Regulate Pulmonary Metastasis of Mammary Carcinomas by Enhancing Protumor Properties of Macrophages, Cancer Cell. (2009) 16, no. 2, 91–102, 10.1016/j.ccr.2009.06.018, 2-s2.0-67651160649. 19647220 PMC2778576

[57] Wang X. , Liu M. , Zhang J. et al., CD24-Siglec Axis is an Innate Immune Checkpoint Against Metaflammation and Metabolic Disorder, Cell Metabolism. (2022) 34, no. 8, 1088–1103, 10.1016/j.cmet.2022.07.005. 35921817 PMC9393047

[58] Liu M. , Li L. , Cao L. et al., Targeted Delivery of CCL3 Reprograms Macrophage Antigen Presentation and Enhances the Efficacy of Immune Checkpoint Blockade Therapy in Hepatocellular Carcinoma, Journal for Immunotherapy of Cancer. (2025) 13, no. 2, 10.1136/jitc-2024-010947. PMC11848677 39988347

[59] Song Q. , Shang J. , Zhang C. , Chen J. , Zhang L. , and Wu X. , Transcription Factor RUNX3 Promotes CD8^+^ T Cell Recruitment by CCL3 and CCL20 in Lung Adenocarcinoma Immune Microenvironment, Journal of Cellular Biochemistry. (2020) 121, no. 5-6, 3208–3220, 10.1002/jcb.29587. 31898342

[60] Liang Y. , Bu Q. , You W. et al., Single-Cell Analysis Reveals Hypoxia-Induced Immunosuppressive Microenvironment in Intrahepatic Cholangiocarcinoma, Biochimica et Biophysica Acta, Molecular Basis of Disease. (2024) 1870, no. 7, 10.1016/j.bbadis.2024.167276. 38844114

[61] Zhang Y. , Lazarus J. , Steele N. G. et al., Regulatory T-Cell Depletion Alters the Tumor Microenvironment and Accelerates Pancreatic Carcinogenesis, Cancer Discovery. (2020) 10, no. 3, 422–439, 10.1158/2159-8290.CD-19-0958. 31911451 PMC7224338

[62] Pitarresi J. R. , Liu X. , Sharma S. M. et al., Stromal ETS2 Regulates Chemokine Production and Immune Cell Recruitment During Acinar-to-Ductal Metaplasia, Neoplasia. (2016) 18, no. 9, 541–552, 10.1016/j.neo.2016.07.006, 2-s2.0-84992420700. 27659014 PMC5031867

[63] Yeh C. R. , Ou Z. Y. , Xiao G. Q. , Guancial E. , and Yeh S. , Infiltrating T Cells Promote Renal Cell Carcinoma (RCC) Progression Via Altering the Estrogen Receptor β-DAB2IP Signals, Oncotarget. (2015) 6, no. 42, 44346–44359, 10.18632/oncotarget.5884, 2-s2.0-84953426240. 26587829 PMC4792561

[64] Kang S. , Xie J. , Ma S. , Liao W. , Zhang J. , and Luo R. , Targeted Knock Down of CCL22 and CCL17 by siRNA During DC Differentiation and Maturation Affects the Recruitment of T Subsets, Immunobiology. (2010) 215, no. 2, 153–162, 10.1016/j.imbio.2009.03.001, 2-s2.0-71849099109. 19450895

[65] Chen J. , Wu Q. , Berglund A. E. , Macaulay R. J. , Mulé J. J. , and Etame A. B. , *RNF135* Expression Marks Chemokine (C-C Motif) Ligand-Enriched Macrophage-Tumor Interactions in the Glioblastoma Microenvironment, Cancers. (2025) 17, no. 19, 10.3390/cancers1719327. PMC12523845 41097797

[66] Xiao Y. , Xu Y. , Wang H. et al., HEBP2-Governed Glutamine Competition Between Tumor and Macrophages Dictates Immunotherapy Efficacy in Triple-Negative Breast Cancer, Cell Metabolism. (2025) 37, no. 10, 2030–2047, 10.1016/j.cmet.2025.08.009. 40992373

[67] To S. K. Y. , Tang M. K. S. , Tong Y. et al., A Selective β-Catenin-Metadherin/CEACAM1-CCL3 Axis Mediates Metastatic Heterogeneity Upon Tumor-Macrophage Interaction, Advanced Science (Weinheim, Baden-Wurttemberg, Germany). (2022) 9, no. 16, 10.1002/advs.202103230. PMC9165500 35403834

[68] Zilio S. , Bicciato S. , Weed D. , and Serafini P. , CCR1 and CCR5 Mediate Cancer-Induced Myelopoiesis and Differentiation of Myeloid Cells in the Tumor, Journal for Immunotherapy of Cancer. (2022) 10, no. 1, 10.1136/jitc-2021-003131. PMC8785210 35064009

[69] Yang Z. , Chu B. , Tu Y. et al., Dual Inhibitors of DNMT and HDAC Remodels the Immune Microenvironment of Colorectal Cancer and Enhances the Efficacy of Anti-PD-L1 Therapy, Pharmacological Research. (2024) 206, 10.1016/j.phrs.2024.107271. 38906202

[70] Xue N. , Lin J. H. , Xing S. et al., Plasma Macrophage Migration Inhibitory Factor and CCL3 as Potential Biomarkers for Distinguishing Patients With Nasopharyngeal Carcinoma From High-Risk Individuals Who Have Positive Epstein-Barr Virus Capsid Antigen-Specific IgA, Cancer Research and Treatment. (2019) 51, no. 1, 378–390, 10.4143/crt.2018.070, 2-s2.0-85059909769. 29807404 PMC6333976

[71] Shcherbina V. , Gordiienko I. , Shlapatska L. , Gluzman D. , and Sidorenko S. , CD150 and CD180 are Negative Regulators of IL-10 Expression and Secretion in Chronic Lymphocytic Leukemia B Cells, Neoplasma. (2021) 68, no. 4, 760–769, 10.4149/neo_2021_210104N8. 33904315

[72] Zhang H. , Wu W. , Wang M. et al., Integrated Peripheral Blood Multi-Omics Profiling Identifies Immune Signatures Predictive of Neoadjuvant PD-1 Blockade Efficacy in Head and Neck Squamous Cell Carcinoma, Journal of Translational Medicine. (2025) 23, no. 1, 10.1186/s12967-025-06770-2. PMC12182656 40544277

[73] Yang J. , Lu X. , Cai Q. et al., Loss of TACC2 Impairs Chemokine CCL3 and CCL4 Expression and Reduces Response to Anti-PD-1 Therapy in Soft Tissue Sarcoma, Molecular Cancer. (2025) 24, no. 1, 10.1186/s12943-025-02354-2. PMC12123857 40442694

[74] Mitchell D. A. , Batich K. A. , Gunn M. D. et al., Tetanus Toxoid and CCL3 Improve Dendritic Cell Vaccines in Mice and Glioblastoma Patients, Nature. (2015) 519, no. 7543, 366–369, 10.1038/nature14320, 2-s2.0-84925045090. 25762141 PMC4510871

[75] González-Martín A. , Gómez L. , Lustgarten J. , Mira E. , and Mañes S. , Maximal T Cell-Mediated Antitumor Responses Rely Upon CCR5 Expression in Both CD4(+) and CD8(+) T Cells, Cancer Research. (2011) 71, no. 16, 5455–5466, 10.1158/0008-5472.CAN-11-1687, 2-s2.0-80051703591. 21715565

